# Supplementary material for: MetaQTL: a package of new computational methods for the meta-analysis of QTL mapping experiments
Source: BMC Bioinformatics. 2007 Feb 8;8:49. doi: 10.1186/1471-2105-8-49 (PMC1808479; doi:10.1186/1471-2105-8-49)
Supplement: Additional File 7 — MetaQTL Package : jar file and tutorial. This Zip archive contains both the MetaQTL JAR file and the files of the tutorial. [file 1471-2105-8-49-S7.zip › org.inra.metaqtl/doc/index-all.html]

 
 
 
 
 
 
Index
 


 

 
function windowTitle()
{
    parent.document.title="Index";
}
 
 
 

 

 


 
   
  
 
 
 
   
 
   
          Overview     
        Package   
        Class   
        Use   
          Tree     
          Deprecated     
       Index    
          Help     
   
 
 
  
 
 
 

 
  
 PREV 
 NEXT  
  
    FRAMES     
   NO FRAMES     
  
    All Classes  ');
  }
  //-->
 
 
    All Classes  
 


  
 
 
  
 

 A   B   C   D   E   F   G   H   I   K   L   M   N   O   P   Q   R   S   T   U   V   W   X   Y   Z   
    
 A  
 
   A2Xml   - Class in  org.metaqtl.main      A2Xml()   - 
Constructor for class org.metaqtl.main. A2Xml 
  
   absolute()   - 
Method in interface org.metaqtl.bio.util. ILGroupPosition 
  
   absolute   - 
Variable in class org.metaqtl.bio.util. LGroupPosition 
  
   absolute()   - 
Method in class org.metaqtl.bio.util. LGroupPosition 
  
   adapt(Chromosome[])   - 
Static method in class org.metaqtl.adapter. MetaQtlAnalysisAdapter 
  
   adapt(EMResult)   - 
Static method in class org.metaqtl.adapter. QtlAdapter 
 From a result of a EM clustering returns the 
 mixture components as an array of Qtl.
   adapt(MetaQtlData)   - 
Static method in class org.metaqtl.adapter. QtlPartitionAdapter 
 Adapt the given   MetaQtlData   into a 
  QtlPartition  and returns it.
   adapt(MetaQtlAnalysis, String, String, int)   - 
Static method in class org.metaqtl.adapter. QtlPartitionAdapter 
 Adapt the   MetaQtlAnalysis    analysis  for
 a given value of the number of clusters  k  and a 
 given trait name  code .
   adapt(MetaQtlResult, Qtl[], int)   - 
Static method in class org.metaqtl.adapter. QtlPartitionAdapter 
  
   addAllele(IBioAllele)   - 
Method in class org.metaqtl.bio.entity. AlleleContainer 
  
   addAllele(IBioAllele)   - 
Method in interface org.metaqtl.bio. IBioLocus 
  
   addAllMap(IBioGenome[])   - 
Method in class org.metaqtl. MapCluster 
  
   addBooleanOption(char, String)   - 
Method in class org.metaqtl.main. CmdLineParser 
 Convenience method for adding a boolean option.
   addBooleanOption(String)   - 
Method in class org.metaqtl.main. CmdLineParser 
 Convenience method for adding a boolean option.
   addChild(TreeNode)   - 
Method in class org.metaqtl. TreeNode 
 Add a child to the node.
   addChromosome(IBioLGroup, IBioCross, int, int, boolean, boolean)   - 
Method in class org.metaqtl. ChromCluster 
 Add a chromosome to the cluster
   addChromosome(IBioLGroup, IBioCross, int, int, boolean, boolean)   - 
Method in class org.metaqtl. MapCluster 
 Add a chromosome to the map cluster framework.
   addDoubleOption(char, String)   - 
Method in class org.metaqtl.main. CmdLineParser 
 Convenience method for adding a double option.
   addDoubleOption(String)   - 
Method in class org.metaqtl.main. CmdLineParser 
 Convenience method for adding a double option.
   addEdge(Chromosome, Chromosome, CMarkerSequence)   - 
Method in class org.metaqtl. ChromCluster.ClusterGraph 
  
   addEntity(IBioEntity)   - 
Method in class org.metaqtl.bio.entity. BioEntityContainer 
  
   addGroup(IBioLGroup)   - 
Method in class org.metaqtl.bio.entity. GroupContainer 
  
   addGroup(IBioLGroup)   - 
Method in interface org.metaqtl.bio. IBioGenome 
  
   addIndividual(IBioIndividual)   - 
Method in class org.metaqtl.bio.entity. IndividualContainer 
  
   addIndividual(IBioIndividual)   - 
Method in interface org.metaqtl.bio. IBioPopulation 
  
   addIntegerOption(char, String)   - 
Method in class org.metaqtl.main. CmdLineParser 
 Convenience method for adding an integer option.
   addIntegerOption(String)   - 
Method in class org.metaqtl.main. CmdLineParser 
 Convenience method for adding an integer option.
   additive   - 
Variable in class org.metaqtl. Qtl.QTLStats 
 The additive effect of the QTL.
   addLocus(IBioLocus)   - 
Method in class org.metaqtl.bio.entity. LocusContainer 
  
   addLocus(IBioLocus)   - 
Method in interface org.metaqtl.bio. IBioLGroup 
  
   addLongOption(char, String)   - 
Method in class org.metaqtl.main. CmdLineParser 
 Convenience method for adding a long integer option.
   addLongOption(String)   - 
Method in class org.metaqtl.main. CmdLineParser 
 Convenience method for adding a long integer option.
   addMap(IBioGenome, boolean)   - 
Method in class org.metaqtl. MapCluster 
 Add a map to the cluster.
   addOption(CmdLineParser.Option)   - 
Method in class org.metaqtl.main. CmdLineParser 
 Add the specified Option to the list of accepted options
   addQtl(Graphics2D, QtlUnit)   - 
Method in class org.metaqtl.graph. QtlLayerBuffer 
 Try to add a qtl unit to the configuration.
   addStringOption(char, String)   - 
Method in class org.metaqtl.main. CmdLineParser 
 Convenience method for adding a string option.
   addStringOption(String)   - 
Method in class org.metaqtl.main. CmdLineParser 
 Convenience method for adding a string option.
   addTerm(String, String)   - 
Method in class org.metaqtl. MetaDico 
  
   adjTable   - 
Variable in class org.metaqtl. ChromCluster.ClusterGraph 
  
   AIC   - 
Static variable in class org.metaqtl. EMCriteria 
  
   aic   - 
Variable in class org.metaqtl. EMCriteria 
  
   AIC3   - 
Static variable in class org.metaqtl. EMCriteria 
  
   aic3   - 
Variable in class org.metaqtl. EMCriteria 
  
   AIC3_NAME   - 
Static variable in class org.metaqtl. EMCriteria 
  
   AIC_NAME   - 
Static variable in class org.metaqtl. EMCriteria 
  
   AICc   - 
Static variable in class org.metaqtl. EMCriteria 
  
   aicc   - 
Variable in class org.metaqtl. EMCriteria 
  
   AICc_NAME   - 
Static variable in class org.metaqtl. EMCriteria 
  
   ALIGN_FIRST_COMMON   - 
Static variable in class org.metaqtl.graph. ChromLayer 
  
   ALIGN_FIRST_MARKER   - 
Static variable in class org.metaqtl.graph. ChromLayer 
  
   alignChromLayers(ChromLayer, ChromLayer, int)   - 
Static method in class org.metaqtl.graph. ChromLayer 
  
   ALLELE   - 
Static variable in interface org.metaqtl.bio. IBioEntityTypes 
 The integer code for Allele entity
   AlleleContainer   - Class in  org.metaqtl.bio.entity  Class Description Here   AlleleContainer()   - 
Constructor for class org.metaqtl.bio.entity. AlleleContainer 
  
   AlleleContainer(String, IBioEntity)   - 
Constructor for class org.metaqtl.bio.entity. AlleleContainer 
  
   alleles()   - 
Method in class org.metaqtl.bio.entity. AlleleContainer 
  
   alleles()   - 
Method in interface org.metaqtl.bio. IBioLocus 
  
   AnchoredElement   - Class in  org.metaqtl.graph      AnchoredElement()   - 
Constructor for class org.metaqtl.graph. AnchoredElement 
  
   areMarkersAroundQTL(int, int, int)   - 
Method in class org.metaqtl. Chromosome 
 Returns true if the QTL position is inside the marker
 interval which markers indices are  mrkIdxL 
 and  mrkIdxR .
   attach(Object)   - 
Method in class org.metaqtl.graph. ChromLayer 
  
   attach(Object)   - 
Method in class org.metaqtl.graph. Layer 
 Attach the given  object  to the layer.
   attach(Object)   - 
Method in class org.metaqtl.graph. QtlLayer 
  
   attach(Object)   - 
Method in class org.metaqtl.graph. QtlPartitionLayer 
  
   attach(Object)   - 
Method in class org.metaqtl.graph. QtlTreeLayer 
  
   attachQtl(Qtl[])   - 
Method in class org.metaqtl. Chromosome 
  
   attachQTL(IBioLocus[])   - 
Method in class org.metaqtl. Chromosome 
 Add
   AWE   - 
Static variable in class org.metaqtl. EMCriteria 
  
   awe   - 
Variable in class org.metaqtl. EMCriteria 
  
   AWE_NAME   - 
Static variable in class org.metaqtl. EMCriteria 
  
 
 
    
 B  
 
   BACKGROUND_COLOR   - 
Static variable in class org.metaqtl.graph. MetaGraphPar 
 The color of the backgroud.
   base   - 
Static variable in class org.metaqtl.graph. PaletteColor 
  
   BC   - 
Static variable in interface org.metaqtl.bio. IBioCrossTypes 
  
   beta(double, double)   - 
Static method in class org.metaqtl.numrec. NumericalUtilities 
 Returns the beta function I(a,b)
   betacf(double, double, double)   - 
Static method in class org.metaqtl.numrec. NumericalUtilities 
 Used by betai : Evaluates continued fraction for incomplete beta function
 by modified Lentz's method.
   betai(double, double, double)   - 
Static method in class org.metaqtl.numrec. NumericalUtilities 
 Returns the incomplete beta function Ix(a,b)
   BIC   - 
Static variable in class org.metaqtl. EMCriteria 
  
   bic   - 
Variable in class org.metaqtl. EMCriteria 
  
   BIC_NAME   - 
Static variable in class org.metaqtl. EMCriteria 
  
   BioCrossTypes   - Class in  org.metaqtl.bio.util      BioCrossTypes()   - 
Constructor for class org.metaqtl.bio.util. BioCrossTypes 
  
   BioEntity   - Class in  org.metaqtl.bio.entity  Class Description Here   BioEntity()   - 
Constructor for class org.metaqtl.bio.entity. BioEntity 
  
   BioEntity(String, IBioEntity)   - 
Constructor for class org.metaqtl.bio.entity. BioEntity 
  
   BioEntityAdapter   - Class in  org.metaqtl.bio.entity.adapter  Class Description Here   BioEntityAdapter()   - 
Constructor for class org.metaqtl.bio.entity.adapter. BioEntityAdapter 
  
   BioEntityBean   - Class in  org.metaqtl.bio.entity.bean  Class Description Here   BioEntityBean()   - 
Constructor for class org.metaqtl.bio.entity.bean. BioEntityBean 
  
   BioEntityCollection   - Class in  org.metaqtl.bio.entity  Class Description Here   BioEntityCollection()   - 
Constructor for class org.metaqtl.bio.entity. BioEntityCollection 
  
   BioEntityCollection(int)   - 
Constructor for class org.metaqtl.bio.entity. BioEntityCollection 
  
   BioEntityCollectionAdapter   - Class in  org.metaqtl.bio.entity.adapter  Class Description Here   BioEntityCollectionAdapter()   - 
Constructor for class org.metaqtl.bio.entity.adapter. BioEntityCollectionAdapter 
  
   BioEntityCollectionBean   - Class in  org.metaqtl.bio.entity.bean  Class Description Here   BioEntityCollectionBean()   - 
Constructor for class org.metaqtl.bio.entity.bean. BioEntityCollectionBean 
  
   BioEntityContainer   - Class in  org.metaqtl.bio.entity  Class Description Here   BioEntityContainer()   - 
Constructor for class org.metaqtl.bio.entity. BioEntityContainer 
  
   BioEntityContainer(String, IBioEntity)   - 
Constructor for class org.metaqtl.bio.entity. BioEntityContainer 
  
   BioEntityFactory   - Class in  org.metaqtl.bio.entity.factory  Class Description Here   BioEntityFactory()   - 
Constructor for class org.metaqtl.bio.entity.factory. BioEntityFactory 
  
   BioEntityNameComparator   - Class in  org.metaqtl.bio.util  Class Description Here   BioLocusTypes   - Class in  org.metaqtl.bio.util      BioLocusTypes()   - 
Constructor for class org.metaqtl.bio.util. BioLocusTypes 
  
   BioUtilities   - Class in  org.metaqtl.bio.util  General comments must be added here.   BioUtilities()   - 
Constructor for class org.metaqtl.bio.util. BioUtilities 
  
   BOX_HEIGHT   - 
Static variable in class org.metaqtl.graph. GraphFactory 
  
   BOX_WIDTH   - 
Static variable in class org.metaqtl.graph. GraphFactory 
  
   build(Graphics2D)   - 
Method in class org.metaqtl.graph. ChromLayer 
  
   build(Graphics2D)   - 
Method in class org.metaqtl.graph. Layer 
 Build the layer.
   build(Graphics2D)   - 
Method in class org.metaqtl.graph. QtlLayer 
  
   build(Graphics2D)   - 
Method in class org.metaqtl.graph. QtlPartitionLayer 
  
   build(Graphics2D)   - 
Method in class org.metaqtl.graph. QtlTreeLayer 
  
   buildMetaChrom(ChromCluster)   - 
Method in class org.metaqtl.algo. MetaMapAlgorithm 
 For a cluster of chromosomes  cluster  this method
 builds the consensus chromosome using a Weighted Least Square
 strategy and returns the result as a   MetaChrom   object.
   buildQtlTree(MetaQtlData)   - 
Method in class org.metaqtl.algo. QtlTreeAlgorithm 
 For the given data points this method build the tree which 
 represents the hierarchical clustering.
   buildShape(Graphics2D, Object)   - 
Method in class org.metaqtl.graph. ChromShape 
  
   buildShape(Graphics2D, Object)   - 
Method in class org.metaqtl.graph. GraphShape 
  
   buildShape(Graphics2D, Object)   - 
Method in class org.metaqtl.graph. QtlShape 
 This methods create a new QtlShape from the given
 graphics context.
 
 
    
 C  
 
   C   - 
Variable in class org.metaqtl.util. OffspringManager.HaldaneWaddingtonProportion 
  
   c1   - 
Variable in class org.metaqtl. Qtl 
 The recombination rate between the QTL position and
 the left flanking marker.
   c2   - 
Variable in class org.metaqtl. Qtl 
 The recombination rate between the QTL position and
 the right flanking marker.
   C_coeff   - 
Static variable in class org.metaqtl.util. OffspringManager.HaldaneWaddingtonProportion 
  
   canAddMoreQtl(double)   - 
Method in class org.metaqtl.graph. QtlLayerBuffer 
 Retruns true if more Qtl can be added to the configuration,
 false otherwise.
   card   - 
Variable in class org.metaqtl. TreeNode 
 The number of sequences in the cluster
   cartoFunction   - 
Static variable in interface org.metaqtl.bio.entity. IGeneticMapProperties 
  
   cartoFunctionHaldane   - 
Static variable in interface org.metaqtl.bio.entity. IGeneticMapProperties 
  
   cartoFunctionKey   - 
Static variable in interface org.metaqtl.bio.entity. IGeneticMapProperties 
  
   cartoFunctionKosambi   - 
Static variable in interface org.metaqtl.bio.entity. IGeneticMapProperties 
  
   ccov   - 
Variable in class org.metaqtl. EMResult 
 The complete information (just for means).
   CENTROID_METHOD   - 
Static variable in class org.metaqtl.algo. HClustAlgorithm 
 Centroid method
   CentroidMethod(double[], double[])   - 
Static method in class org.metaqtl.algo. HClustAlgorithm 
 This method performs a hierarchical clustering
 of the given data points using a centroid method
 based on the mahalanobis distance.
   cg   - 
Variable in class org.metaqtl. Chromosome 
 The number of generations of the offspring population.
   checkProjection(ChromCluster[])   - 
Method in class org.metaqtl.algo. QtlProjAlgorithm 
  
   chi2   - 
Variable in class org.metaqtl. MetaChrom 
 The chi-square value of this chromosome.
   chi2   - 
Variable in class org.metaqtl. MultiFitResult 
 The chi-square of the model.
   children   - 
Variable in class org.metaqtl.bio.entity.bean. OntologyTermBean 
 The children of the term.
   children   - 
Variable in class org.metaqtl. TreeNode 
 Left and right children, or null
   CHISQUARE   - 
Static variable in interface org.metaqtl. IBioLGroupProperties 
 The key for the chisquare value.
   chrName   - 
Variable in class org.metaqtl.algo. ClustAlgorithm 
 The name of the chromosome to study.
   chrom   - 
Variable in class org.metaqtl. ChromCluster.ClusterEdge 
  
   CHROM_ALIGN_MODE   - 
Static variable in class org.metaqtl.graph. MetaGraphPar 
  
   CHROM_DISTANCE_SCALE   - 
Static variable in class org.metaqtl.graph. MetaGraphPar 
  
   CHROM_FLANKING_CEX   - 
Static variable in class org.metaqtl.graph. MetaGraphPar 
 The default cex parameter for flanking regions at the 
 top and the end of the chromosome.
   CHROM_NAME_FONT   - 
Static variable in class org.metaqtl.graph. MetaGraphPar 
  
   CHROM_NAME_HSPACE   - 
Static variable in class org.metaqtl.graph. MetaGraphPar 
  
   CHROM_STROKE   - 
Static variable in class org.metaqtl.graph. MetaGraphPar 
 The stroke of the chromosome shape.
   CHROM_STROKE_COLOR   - 
Static variable in class org.metaqtl.graph. MetaGraphPar 
 The color of the stroke of the Chromosome shape.
   CHROM_TICK_COLOR   - 
Static variable in class org.metaqtl.graph. MetaGraphPar 
 The color of the marker ticks on the chromosome.
   CHROM_TICK_STROKE   - 
Static variable in class org.metaqtl.graph. MetaGraphPar 
  
   CHROM_TICK_WIDTH_1   - 
Static variable in class org.metaqtl.graph. MetaGraphPar 
 The width of the marker ticks.
   CHROM_TICK_WIDTH_2   - 
Static variable in class org.metaqtl.graph. MetaGraphPar 
  
   CHROM_TICK_WIDTH_3   - 
Static variable in class org.metaqtl.graph. MetaGraphPar 
  
   CHROM_WIDTH   - 
Static variable in class org.metaqtl.graph. MetaGraphPar 
 The width of the chromosome.
   ChromAxe   - Class in  org.metaqtl.graph      ChromAxe(double, double, double)   - 
Constructor for class org.metaqtl.graph. ChromAxe 
  
   ChromCluster   - Class in  org.metaqtl  A cluster of chromosomes which share the same name inside a 
   MapCluster   framwork.   ChromCluster()   - 
Constructor for class org.metaqtl. ChromCluster 
 Creates a new instance if ChromCluster.
   ChromCluster.ClusterEdge   - Class in  org.metaqtl      ChromCluster.ClusterEdge(CMarkerSequence, Chromosome)   - 
Constructor for class org.metaqtl. ChromCluster.ClusterEdge 
  
   ChromCluster.ClusterGraph   - Class in  org.metaqtl      ChromCluster.ClusterGraph(ChromCluster)   - 
Constructor for class org.metaqtl. ChromCluster.ClusterGraph 
  
   ChromGraph   - Class in  org.metaqtl.graph  A  ChromGraph  allows to link together
 a  Chromosome  and a  QtlPartition 
 in order to be depicted into a graphic.   ChromGraph()   - 
Constructor for class org.metaqtl.graph. ChromGraph 
  
   ChromLayer   - Class in  org.metaqtl.graph      ChromLayer(double, double)   - 
Constructor for class org.metaqtl.graph. ChromLayer 
  
   ChromLayer.CMrkTickPath   - Class in  org.metaqtl.graph      ChromLayer.CMrkTickPath()   - 
Constructor for class org.metaqtl.graph. ChromLayer.CMrkTickPath 
  
   ChromLayer.CMrkTickPoint   - Class in  org.metaqtl.graph      ChromLayer.CMrkTickPoint()   - 
Constructor for class org.metaqtl.graph. ChromLayer.CMrkTickPoint 
  
   chromNames   - 
Variable in class org.metaqtl. MetaQtlAnalysis 
 The names of the chromosomes.
   chromNames   - 
Variable in class org.metaqtl. MetaQtlModel 
 The chromosomes.
   Chromosome   - Class in  org.metaqtl  On a Chromosome there are two types of locus :
 
  Marker 
  QTL (Quantitative Trait Loci) 
 
 inside a mapping context which is defined by both the mapping framework
 (function and unit) and the population cross design used to build this
 chromosome.   Chromosome()   - 
Constructor for class org.metaqtl. Chromosome 
  
   chromosome   - 
Variable in class org.metaqtl.graph. ChromGraph 
 The chromosome to draw
   ChromosomeAdapter   - Class in  org.metaqtl.adapter  This class defines some usefull methods to adapt   Chromosome  
 objects into other kinds of objects or the inverse.   ChromosomeAdapter()   - 
Constructor for class org.metaqtl.adapter. ChromosomeAdapter 
  
   chromosomes   - 
Variable in class org.metaqtl.algo. ClustAlgorithm 
 The chromosome on which the clustering is performed.
   chromosomes   - 
Variable in class org.metaqtl. MetaMap 
 The meta chromosomes.
   ChromShape   - Class in  org.metaqtl.graph      ChromShape()   - 
Constructor for class org.metaqtl.graph. ChromShape 
  
   ci   - 
Variable in class org.metaqtl. Qtl 
 The QTL confidence interval.
   ci2sd(double)   - 
Static method in class org.metaqtl.numrec. NumericalUtilities 
  
   ci_height   - 
Variable in class org.metaqtl.graph. QtlShape 
  
   CI_MISS_IGNORE   - 
Static variable in interface org.metaqtl. IMetaQtlConstants 
 MSD Mode : ignore QTL with missing standard deviation.
   CI_MISS_IMPUTATION   - 
Static variable in interface org.metaqtl. IMetaQtlConstants 
 MSD Mode : replace missing standard deviation
 by the mean of the observed ones.
   CI_MODE_AVAILABLE   - 
Static variable in interface org.metaqtl. IMetaQtlConstants 
 SD Mode : use the available information to compute the 
 standard deviation.
   CI_MODE_LOD   - 
Static variable in interface org.metaqtl. IMetaQtlConstants 
 SD Mode : use only the confidence interval information
 if defined to compute the standard deviation.
   CI_MODE_MAXIMAL   - 
Static variable in interface org.metaqtl. IMetaQtlConstants 
 SD Mode : compute the standard deviation using
 both lod support and rsquare information (if available) 
 and then choose the maximal standard-deviation.
   CI_MODE_RSQUARE   - 
Static variable in interface org.metaqtl. IMetaQtlConstants 
 SD Mode : use only the rsquare information
 if defined to compute the standard deviation.
   CI_WIDTH   - 
Static variable in class org.metaqtl.graph. QtlLayer 
 The default width of the CI of the Qtl
   ci_width   - 
Variable in class org.metaqtl.graph. QtlShape 
  
   clog   - 
Variable in class org.metaqtl. EMResult 
 The complete loglikelihood.
   close()   - 
Method in class org.metaqtl.bio.util. TabulatedReader 
 Closes the stream.
   close()   - 
Method in class org.metaqtl.bio.util. TabulatedWriter 
  
   ClustAlgorithm   - Class in  org.metaqtl.algo      ClustAlgorithm(IBioGenome, IBioOntology)   - 
Constructor for class org.metaqtl.algo. ClustAlgorithm 
  
   ClustAlgorithm(IBioGenome, String, IBioOntology)   - 
Constructor for class org.metaqtl.algo. ClustAlgorithm 
  
   cluster   - 
Variable in class org.metaqtl. ChromCluster.ClusterGraph 
  
   clusterings   - 
Variable in class org.metaqtl. MetaQtlResult 
 The meta qtl clusterings.
   clusters   - 
Variable in class org.metaqtl. ChromCluster 
 The list of the cluster members.
   CMarkerSequence   - Class in  org.metaqtl  A Common marker sequence between two ordered set of markers
 is a sub-set of at least two markers which order is the same between
 the two sets (may be reverse).   CMarkerSequence(int[][], int)   - 
Constructor for class org.metaqtl. CMarkerSequence 
 Initiates a new Common marker sequence from the
 tow arrays of common marker indices
  mcidx[0..1][0..nmc-1] .
   CmdLineParser   - Class in  org.metaqtl.main  Largely GNU-compatible command-line options parser.   CmdLineParser()   - 
Constructor for class org.metaqtl.main. CmdLineParser 
  
   CmdLineParser.IllegalOptionValueException   - Exception in  org.metaqtl.main  Thrown when an illegal or missing value is given by the user for
 an option that takes a value.   CmdLineParser.IllegalOptionValueException(CmdLineParser.Option, String)   - 
Constructor for exception org.metaqtl.main. CmdLineParser.IllegalOptionValueException 
  
   CmdLineParser.NotFlagException   - Exception in  org.metaqtl.main  Thrown when the parsed commandline contains multiple concatenated
 short options, such as -abcd, where one or more requires a value.   CmdLineParser.NotFlagException(String, char)   - 
Constructor for exception org.metaqtl.main. CmdLineParser.NotFlagException 
  
   CmdLineParser.Option   - Class in  org.metaqtl.main  Representation of a command-line option   CmdLineParser.Option(String, boolean)   - 
Constructor for class org.metaqtl.main. CmdLineParser.Option 
  
   CmdLineParser.Option(char, String, boolean)   - 
Constructor for class org.metaqtl.main. CmdLineParser.Option 
  
   CmdLineParser.Option.BooleanOption   - Class in  org.metaqtl.main      CmdLineParser.Option.BooleanOption(char, String)   - 
Constructor for class org.metaqtl.main. CmdLineParser.Option.BooleanOption 
  
   CmdLineParser.Option.BooleanOption(String)   - 
Constructor for class org.metaqtl.main. CmdLineParser.Option.BooleanOption 
  
   CmdLineParser.Option.DoubleOption   - Class in  org.metaqtl.main  An option that expects a floating-point value   CmdLineParser.Option.DoubleOption(char, String)   - 
Constructor for class org.metaqtl.main. CmdLineParser.Option.DoubleOption 
  
   CmdLineParser.Option.DoubleOption(String)   - 
Constructor for class org.metaqtl.main. CmdLineParser.Option.DoubleOption 
  
   CmdLineParser.Option.IntegerOption   - Class in  org.metaqtl.main  An option that expects an integer value   CmdLineParser.Option.IntegerOption(char, String)   - 
Constructor for class org.metaqtl.main. CmdLineParser.Option.IntegerOption 
  
   CmdLineParser.Option.IntegerOption(String)   - 
Constructor for class org.metaqtl.main. CmdLineParser.Option.IntegerOption 
  
   CmdLineParser.Option.LongOption   - Class in  org.metaqtl.main  An option that expects a long integer value   CmdLineParser.Option.LongOption(char, String)   - 
Constructor for class org.metaqtl.main. CmdLineParser.Option.LongOption 
  
   CmdLineParser.Option.LongOption(String)   - 
Constructor for class org.metaqtl.main. CmdLineParser.Option.LongOption 
  
   CmdLineParser.Option.StringOption   - Class in  org.metaqtl.main  An option that expects a string value   CmdLineParser.Option.StringOption(char, String)   - 
Constructor for class org.metaqtl.main. CmdLineParser.Option.StringOption 
  
   CmdLineParser.Option.StringOption(String)   - 
Constructor for class org.metaqtl.main. CmdLineParser.Option.StringOption 
  
   CmdLineParser.OptionException   - Exception in  org.metaqtl.main  Base class for exceptions that may be thrown when options are parsed   CmdLineParser.OptionException(String)   - 
Constructor for exception org.metaqtl.main. CmdLineParser.OptionException 
  
   CmdLineParser.UnknownOptionException   - Exception in  org.metaqtl.main  Thrown when the parsed command-line contains an option that is not
 recognised.   CmdLineParser.UnknownOptionException(String)   - 
Constructor for exception org.metaqtl.main. CmdLineParser.UnknownOptionException 
  
   CmdLineParser.UnknownOptionException(String, String)   - 
Constructor for exception org.metaqtl.main. CmdLineParser.UnknownOptionException 
  
   CmdLineParser.UnknownSuboptionException   - Exception in  org.metaqtl.main  Thrown when the parsed commandline contains multiple concatenated
 short options, such as -abcd, where one is unknown.   CmdLineParser.UnknownSuboptionException(String, char)   - 
Constructor for exception org.metaqtl.main. CmdLineParser.UnknownSuboptionException 
  
   cms   - 
Variable in class org.metaqtl. ChromCluster.ClusterEdge 
  
   CMSAlgorithm   - Class in  org.metaqtl.algo  Use this class to find common marker sequences between
 two chromosomes.   CMSAlgorithm()   - 
Constructor for class org.metaqtl.algo. CMSAlgorithm 
  
   COLLECTION   - 
Static variable in interface org.metaqtl.bio. IBioEntityTypes 
  
   color2string(Color)   - 
Static method in class org.metaqtl.graph. MetaGraphPar 
  
   COMMON_STROKE_WIDTH   - 
Static variable in class org.metaqtl.graph. MetaGraphPar 
  
   compare(Object, Object)   - 
Method in class org.metaqtl.bio.util. BioEntityNameComparator 
  
   compare(Object, Object)   - 
Method in class org.metaqtl.bio.util. LocusPositionComparator 
  
   computeCOV(double[], EMResult)   - 
Static method in class org.metaqtl.algo. EMAlgorithm 
 Computes the variance-covariance matrix of the 
 mixture component estimates.
   computeCriteria()   - 
Method in class org.metaqtl. EMResult 
 This methos computes the following criteria :
 
  AIC 
  AIC3 
  ICOMP 
  MIR   
  BIC 
  AWE 
 
   computeDM(double[], double[], double[], EMResult)   - 
Static method in class org.metaqtl.algo. EMAlgorithm 
 Computes the matrix of derivatives of the update
 function.
   computeQtlSD(int)   - 
Method in class org.metaqtl. Chromosome 
 According to the given standard deviation computation
 mode this method computes for each QTL the value of the standard
 deviation of the QTL position.
   ConsMap   - Class in  org.metaqtl.main      ConsMap()   - 
Constructor for class org.metaqtl.main. ConsMap 
  
   containsKey(String)   - 
Method in class org.metaqtl.bio.util. TabulatedReader 
 Returns true if the given string is a key of the tabulated file.
   copy(EMResult, EMResult)   - 
Static method in class org.metaqtl. EMResult 
  
   cov   - 
Variable in class org.metaqtl. MultiFitResult 
 The variance-covariance matrix of the parameter
 estimates.
   criteria   - 
Variable in class org.metaqtl. EMResult 
 The model choice criteria.
   cross   - 
Variable in class org.metaqtl. Qtl 
 The QTL cross design.
   CROSS_SIZE   - 
Static variable in interface org.metaqtl. IBioGenomeProperties 
 The cross size key.
   CROSS_TYPE   - 
Static variable in interface org.metaqtl. IBioGenomeProperties 
 The cross type key.
   crossLayers   - 
Variable in class org.metaqtl.graph. MetaGraph 
  
   CrossPopulation   - Class in  org.metaqtl.bio.entity  Class Description Here   CrossPopulation()   - 
Constructor for class org.metaqtl.bio.entity. CrossPopulation 
  
   CrossPopulation(String, IBioEntity)   - 
Constructor for class org.metaqtl.bio.entity. CrossPopulation 
  
   CrossPopulation(String, IBioEntity, int)   - 
Constructor for class org.metaqtl.bio.entity. CrossPopulation 
  
   crossToString(int, int)   - 
Static method in class org.metaqtl.bio.util. BioCrossTypes 
  
   cs   - 
Variable in class org.metaqtl. Chromosome 
 The size of the offspring population.
   css   - 
Variable in class org.metaqtl. CMarkerSequence 
 The sizes of the common sequences
   ct   - 
Variable in class org.metaqtl. Chromosome 
 The type of the offspring population.
 
 
    
 D  
 
   d   - 
Variable in class org.metaqtl. MetaChrom 
 The absolute positions of markers in Morgan.
   D   - 
Variable in class org.metaqtl.util. OffspringManager.HaldaneWaddingtonProportion 
  
   D_coeff   - 
Static variable in class org.metaqtl.util. OffspringManager.HaldaneWaddingtonProportion 
  
   dataLayers   - 
Variable in class org.metaqtl.graph. MetaGraph 
  
   dC   - 
Variable in class org.metaqtl.util. OffspringManager.HaldaneWaddingtonProportion 
  
   dD   - 
Variable in class org.metaqtl.util. OffspringManager.HaldaneWaddingtonProportion 
  
   dE   - 
Variable in class org.metaqtl.util. OffspringManager.HaldaneWaddingtonProportion 
  
   DEFAULT_HEIGHT   - 
Static variable in class org.metaqtl.graph. MetaGraphPar 
 The default height.
   DEFAULT_WIDTH   - 
Static variable in class org.metaqtl.graph. MetaGraphPar 
 The default width.
   DEFINITION   - 
Static variable in interface org.metaqtl.bio. IOntologyTermProperties 
 The definition property of a term in a ontology
   dF   - 
Variable in class org.metaqtl.util. OffspringManager.HaldaneWaddingtonProportion 
  
   dfridr(NumericalUtilities.Function, double, double)   - 
Static method in class org.metaqtl.numrec. NumericalUtilities 
 Returns the derivative of a function func at a point x by Ridders' method of
 polynomial extrapolation.
   dG   - 
Variable in class org.metaqtl.util. OffspringManager.HaldaneWaddingtonProportion 
  
   DH   - 
Static variable in interface org.metaqtl.bio. IBioCrossTypes 
  
   disableLogger()   - 
Method in class org.metaqtl.algo. MetaAlgorithm 
  
   dist   - 
Variable in class org.metaqtl. TreeNode 
 The distance between the node and its parent.
   distance(double, int, int)   - 
Static method in class org.metaqtl.util. MappingFunction 
 Returns the distance from the recombination rate.
   distanceUnitCM   - 
Static variable in interface org.metaqtl.bio.entity. IGeneticMapProperties 
  
   distanceUnitKey   - 
Static variable in interface org.metaqtl.bio.entity. IGeneticMapProperties 
  
   dm   - 
Variable in class org.metaqtl. EMResult 
 The matrix of convergence rate
 for the EM-Algorithm (just for means).
   DMAX(double, double)   - 
Static method in class org.metaqtl.numrec. NumericalUtilities 
 Returns the max between  a  and  b 
   DO_SEM   - 
Static variable in class org.metaqtl.algo. EMAlgorithm 
 If true the best starting point is used to
 do Supplemental EM (SEM) in order to
 compute the DM Matrix..
   doEM(double[], double[], int, EMResult)   - 
Static method in class org.metaqtl.algo. EMAlgorithm 
 Apply the EM-Algorithm on the given data set where  x 
 is an array of observed value and  sd  an array of same
 size than  x  which stores the standard deviations of 
 the observed values.
   DOF   - 
Static variable in interface org.metaqtl. IBioLGroupProperties 
 The key for the number of degrees of freedom.
   dof   - 
Variable in class org.metaqtl. MetaChrom 
 The number of degrees of freedom.
   dof   - 
Variable in class org.metaqtl. MultiFitResult 
 The number of degrees of freedom.
   dominance   - 
Variable in class org.metaqtl. Qtl.QTLStats 
 The dominance effect of the QTL.
   doProjection(ChromCluster[])   - 
Method in class org.metaqtl.algo. QtlProjAlgorithm 
  
   doQtlClustering(MetaQtlData)   - 
Method in class org.metaqtl.algo. QtlClustAlgorithm 
 For a given data set  data  performs the clustering
 on each trait group and returns the results as an array of 
   MetaQtlResult   objects.
   doTraitGroupClustering()   - 
Method in class org.metaqtl. MetaQtlData 
 According to the values of the QTLTrait.group
 attribute of the trait attribute of the qtls
 this methos clusterize the qtls in trait
 groups.
   doTraitOntologyClustering(IBioOntology)   - 
Method in class org.metaqtl. MetaQtlData 
 According to the given trait ontology this methods
 clusterize the QTL in group in such a way that each
 group represents a trait term in the ontology.
   draw(Graphics2D)   - 
Method in class org.metaqtl.graph. ChromLayer 
  
   draw(Graphics2D)   - 
Method in class org.metaqtl.graph. ChromShape 
  
   draw(Graphics2D)   - 
Method in class org.metaqtl.graph. GraphShape 
 Draws the shape.
   draw(Graphics2D)   - 
Method in class org.metaqtl.graph. Layer 
 Draws the layer on the given  graph  context.
   draw(Graphics2D, double, double)   - 
Method in class org.metaqtl.graph. MetaGraph 
  
   draw(Graphics2D)   - 
Method in class org.metaqtl.graph. QtlLayer 
  
   draw(Graphics2D)   - 
Method in class org.metaqtl.graph. QtlPartitionLayer 
  
   draw(Graphics2D)   - 
Method in class org.metaqtl.graph. QtlShape 
  
   draw(Graphics2D)   - 
Method in class org.metaqtl.graph. QtlTreeLayer 
  
   DSWAP(double[], int, int)   - 
Static method in class org.metaqtl.numrec. NumericalUtilities 
 Swaps elements i and j in a.
   DUBIOUS_ORDER   - 
Static variable in interface org.metaqtl. IBioLocusProperties 
 The key for the marker dubious status.
   DubiousLocusFactory   - Class in  org.metaqtl.factory  A factory to read a list of dubious markers from a file.   DubiousLocusFactory()   - 
Constructor for class org.metaqtl.factory. DubiousLocusFactory 
  
 
 
    
 E  
 
   E   - 
Variable in class org.metaqtl.util. OffspringManager.HaldaneWaddingtonProportion 
  
   E_coeff   - 
Static variable in class org.metaqtl.util. OffspringManager.HaldaneWaddingtonProportion 
  
   edist   - 
Variable in class org.metaqtl. EMResult 
 The euclidean distance between the 
 current and the previous vector of
 parameters (mu + pi)
   EM_CONTINUE   - 
Static variable in class org.metaqtl.algo. EMAlgorithm 
 The EM convergence status : continue
   EM_ERR   - 
Static variable in class org.metaqtl.algo. EMAlgorithm 
 The EM convergence tolerance.
   EM_FAILURE   - 
Static variable in class org.metaqtl.algo. EMAlgorithm 
 The EM convergence status : failure
   EM_ITER_MAX   - 
Static variable in class org.metaqtl.algo. EMAlgorithm 
 The EM maximum number of iterations.
   EM_MIN_DISTANCE   - 
Static variable in class org.metaqtl.algo. EMAlgorithm 
 The minimal mahalanobis distance between
 components.
   EM_OK   - 
Static variable in class org.metaqtl.algo. EMAlgorithm 
 The EM convergence status : success
   EM_START   - 
Static variable in class org.metaqtl.algo. EMAlgorithm 
 The number of replicates for a run of the EM-algorithm.
   EMAlgorithm   - Class in  org.metaqtl.algo  This class defines methods to perform the EM-algorithm
 for univariate mixture of gaussians with known variances.   EMAlgorithm()   - 
Constructor for class org.metaqtl.algo. EMAlgorithm 
  
   EMCriteria   - Class in  org.metaqtl      EMCriteria()   - 
Constructor for class org.metaqtl. EMCriteria 
  
   EMPTY_BIOALLELE_ARRAY   - 
Static variable in interface org.metaqtl.bio.entity. IBioConstants 
  
   EMPTY_BIOENTITY_ARRAY   - 
Static variable in interface org.metaqtl.bio.entity. IBioConstants 
  
   EMPTY_BIOLGROUP_ARRAY   - 
Static variable in interface org.metaqtl.bio.entity. IBioConstants 
  
   EMPTY_BIOLOCUS_ARRAY   - 
Static variable in interface org.metaqtl.bio.entity. IBioConstants 
  
   emRate(EMResult, double[], double[])   - 
Static method in class org.metaqtl.algo. EMAlgorithm 
 Compute the euclidean distance between the new parameters and the
 old ones to obtain a simple approximation of the EM convergence
 rate.
   EMResult   - Class in  org.metaqtl      EMResult()   - 
Constructor for class org.metaqtl. EMResult 
  
   EMResult(int, int)   - 
Constructor for class org.metaqtl. EMResult 
  
   EMResultFactory   - Class in  org.metaqtl.factory      EMResultFactory()   - 
Constructor for class org.metaqtl.factory. EMResultFactory 
  
   entities   - 
Variable in class org.metaqtl.bio.entity.bean. BioEntityCollectionBean 
  
   entities   - 
Variable in class org.metaqtl.bio.entity. BioEntityContainer 
  
   entities()   - 
Method in class org.metaqtl.bio.entity. BioEntityContainer 
  
   entityNumber()   - 
Method in class org.metaqtl.bio.entity. BioEntityContainer 
  
   eStep(double[], double[], EMResult)   - 
Static method in class org.metaqtl.algo. EMAlgorithm 
 The Expectation step : it just consists in updating the 
 Z matrix, i.e the cluster membership probabilities.
   ESTIMATED   - 
Static variable in interface org.metaqtl.bio.util. ILGroupPosition 
  
   EstimatedLocusPosition   - Class in  org.metaqtl.bio.util  Class Description Here   EstimatedLocusPosition(double, double)   - 
Constructor for class org.metaqtl.bio.util. EstimatedLocusPosition 
  
   extractLocus(IBioLocus[], int)   - 
Static method in class org.metaqtl.bio.util. BioLocusTypes 
 This returns extracts in the given array of loci
 the locus which type matches the given one.
 
 
    
 F  
 
   F   - 
Variable in class org.metaqtl.util. OffspringManager.HaldaneWaddingtonProportion 
  
   F_coeff   - 
Static variable in class org.metaqtl.util. OffspringManager.HaldaneWaddingtonProportion 
  
   fisherVariance(double, int, int, int)   - 
Static method in class org.metaqtl.util. OffspringManager 
 Returns the inverse of the Fisher Information depending on
 the type of cross for a given recombination rate and a given
 population size.
   fixCluster()   - 
Method in class org.metaqtl. ChromCluster 
 This methods frozes the index of the distinct
 markers over all the members of the cluster.
   font2string(Font)   - 
Static method in class org.metaqtl.graph. MetaGraphPar 
  
   formatDouble(double)   - 
Static method in class org.metaqtl.bio.util. NumberFormat 
  
   frames   - 
Variable in class org.metaqtl. CMarkerSequence 
 The frame of the common marker sequences
   from   - 
Variable in class org.metaqtl.graph. QtlUnit 
  
   from   - 
Variable in class org.metaqtl. Qtl.QTLSupport 
 The position from the first marker
 to the left border of the IC.
   fromEntity(IBioEntity, BioEntityBean)   - 
Static method in class org.metaqtl.bio.entity.adapter. BioEntityAdapter 
  
   fromEntity(IBioEntity)   - 
Method in class org.metaqtl.bio.entity.adapter. BioEntityCollectionAdapter 
  
   fromEntity(IBioEntity)   - 
Method in class org.metaqtl.bio.entity.adapter. GeneticMapBeanAdapter 
  
   fromEntity(IBioEntity)   - 
Method in class org.metaqtl.bio.entity.adapter. LGroupBeanAdapter 
  
   fromEntity(IBioEntity)   - 
Method in class org.metaqtl.bio.entity.adapter. LocusBeanAdapter 
  
   fromEntity(IBioEntity)   - 
Method in class org.metaqtl.bio.entity.adapter. OntologyBeanAdapter 
  
   fromEntity(IBioEntity)   - 
Method in class org.metaqtl.bio.entity.adapter. OntologyTermBeanAdapter 
  
   fromEntity(IBioEntity)   - 
Method in interface org.metaqtl.bio. IBioAdapter 
 Convert the given compliant object to a
 bio entity object.
   fromQTL(Qtl)   - 
Static method in class org.metaqtl.adapter. QtlAdapter 
 This method converts a   Qtl   into an object which class
 implements the  IBioLocus  interface.
   func(double)   - 
Method in interface org.metaqtl.numrec. NumericalUtilities.Function 
 Returns the value of the function in x.
   funcd(double)   - 
Method in interface org.metaqtl.numrec. NumericalUtilities.NRFunction 
 Compute both the value and the derivate of the
 function in x.
   function   - 
Variable in class org.metaqtl.bio.entity.bean. OntologyBean 
 The function of the ontology.
 
 
    
 G  
 
   G   - 
Variable in class org.metaqtl.util. OffspringManager.HaldaneWaddingtonProportion 
  
   G_coeff   - 
Static variable in class org.metaqtl.util. OffspringManager.HaldaneWaddingtonProportion 
  
   GAMCOF   - 
Static variable in class org.metaqtl.numrec. NumericalUtilities 
  
   gammln(double)   - 
Static method in class org.metaqtl.numrec. NumericalUtilities 
  
   gammp(double, double)   - 
Static method in class org.metaqtl.numrec. NumericalUtilities 
  
   gauss(double)   - 
Static method in class org.metaqtl.numrec. NumericalUtilities 
 This routine returns the value at x of the normal
 centered gaussian density.
   gcf(double, double)   - 
Static method in class org.metaqtl.numrec. NumericalUtilities 
  
   gen   - 
Static variable in class org.metaqtl.bio.util. BioCrossTypes 
  
   gen   - 
Variable in class org.metaqtl. Qtl.QTLCross 
 The number of generations.
   generalHelp()   - 
Static method in class org.metaqtl.main. MetaMain 
  
   generalUsage()   - 
Static method in class org.metaqtl.main. MetaMain 
  
   generation   - 
Variable in class org.metaqtl.bio.entity. Population 
  
   GeneticMap   - Class in  org.metaqtl.bio.entity  Class Description Here   GeneticMap()   - 
Constructor for class org.metaqtl.bio.entity. GeneticMap 
  
   GeneticMap(String, IBioEntity)   - 
Constructor for class org.metaqtl.bio.entity. GeneticMap 
  
   GeneticMap(String)   - 
Constructor for class org.metaqtl.bio.entity. GeneticMap 
  
   GeneticMapBean   - Class in  org.metaqtl.bio.entity.bean  Class Description Here   GeneticMapBean()   - 
Constructor for class org.metaqtl.bio.entity.bean. GeneticMapBean 
  
   GeneticMapBeanAdapter   - Class in  org.metaqtl.bio.entity.adapter  Class Description Here   GeneticMapBeanAdapter()   - 
Constructor for class org.metaqtl.bio.entity.adapter. GeneticMapBeanAdapter 
  
   GeneticMapProperties   - Class in  org.metaqtl.util  General comments must be added here.   GeneticMapProperties()   - 
Constructor for class org.metaqtl.util. GeneticMapProperties 
  
   GENOME   - 
Static variable in interface org.metaqtl.bio. IBioEntityTypes 
 The integer code for Genome entity
   get(String)   - 
Method in class org.metaqtl.bio.util. TabulatedReader 
 Returns the value of the field which belongs to the given key.
   getAdjacentTable()   - 
Method in class org.metaqtl. ChromCluster.ClusterGraph 
  
   getAllele(String)   - 
Method in class org.metaqtl.bio.entity. AlleleContainer 
  
   getAllele(String)   - 
Method in interface org.metaqtl.bio. IBioLocus 
  
   getAlleleNumber()   - 
Method in class org.metaqtl.bio.entity. AlleleContainer 
  
   getAlleleNumber()   - 
Method in interface org.metaqtl.bio. IBioLocus 
  
   getBestClustering(int)   - 
Method in class org.metaqtl. MetaQtlResult 
 This methods returns the best models for each criterion
 defined to select the optimal number of clusters per
 trait.
   getBestModel(int)   - 
Method in class org.metaqtl. MetaQtlAnalysis 
 This methods returns the best models for each criterion
 defined to select the optimal number of clusters per
 chromosome per trait.
   getBestResult(EMResult[], int)   - 
Static method in class org.metaqtl. EMCriteria 
 For the given criterion  criterion  this methods
 looks into the array of   EMResult   and find the model
 which is optimal.
   getBestResult(EMResult[], int, int)   - 
Static method in class org.metaqtl. EMCriteria 
  
   getBestResult(EMResult[], String)   - 
Static method in class org.metaqtl. EMCriteria 
  
   getBestResult(EMResult[], int, String)   - 
Static method in class org.metaqtl. EMCriteria 
  
   getBioAdapter()   - 
Method in class org.metaqtl.bio.entity. BioEntityCollection 
  
   getBioAdapter()   - 
Method in class org.metaqtl.bio.entity. GeneticMap 
  
   getBioAdapter()   - 
Method in class org.metaqtl.bio.entity. Individual 
  
   getBioAdapter()   - 
Method in class org.metaqtl.bio.entity. LGroup 
  
   getBioAdapter()   - 
Method in class org.metaqtl.bio.entity. Marker 
  
   getBioAdapter()   - 
Method in class org.metaqtl.bio.entity. Ontology 
  
   getBioAdapter()   - 
Method in class org.metaqtl.bio.entity. OntologyTerm 
  
   getBioAdapter()   - 
Method in class org.metaqtl.bio.entity. Population 
  
   getBioAdapter()   - 
Method in class org.metaqtl.bio.entity. QTL 
  
   getBioAdapter()   - 
Method in interface org.metaqtl.bio. IBioAdaptable 
  
   getCard()   - 
Method in class org.metaqtl. TreeNode 
  
   getCentroid(double[], double[])   - 
Method in class org.metaqtl. TreeNode 
  
   getChildren()   - 
Method in class org.metaqtl. TreeNode 
  
   getChildrenTerm()   - 
Method in class org.metaqtl.bio.entity. OntologyTerm 
  
   getChildrenTerm()   - 
Method in interface org.metaqtl.bio. IBioOntologyTerm 
 Returns the children if the term has chilren,
 null otherwise.
   getChromAxe()   - 
Method in class org.metaqtl.graph. ChromLayer 
 Return the axe of the chromosome.
   getChromAxe()   - 
Method in class org.metaqtl.graph. ChromShape 
  
   getChromAxe()   - 
Method in class org.metaqtl.graph. QtlPartitionLayer 
  
   getChromByNames(Chromosome[], String[])   - 
Static method in class org.metaqtl.main. MetaMain 
  
   getChromGraph(Chromosome[], boolean, int, IBioOntology, MetaQtlAnalysis)   - 
Static method in class org.metaqtl.main. MetaMain 
  
   getChromIdx(String)   - 
Method in class org.metaqtl. MetaQtlAnalysis 
  
   getChromNames()   - 
Method in class org.metaqtl. MetaQtlModel 
 Returns the names of the chromosomes.
   getChromosomeName(String)   - 
Method in class org.metaqtl. MapCluster 
 This method returns the name of the chromosome
 from the given name.
   getChromWidth()   - 
Method in class org.metaqtl.graph. ChromLayer 
  
   getChromWidth()   - 
Method in class org.metaqtl.graph. ChromShape 
  
   getCluster(String)   - 
Static method in class org.metaqtl.main. MetaMain 
  
   getClusterByNames(ChromCluster[], String[])   - 
Static method in class org.metaqtl.main. MetaMain 
  
   getClustering(int)   - 
Method in class org.metaqtl. MetaQtlResult 
 Returns the Qtl clustering result with  k 
 clusters.
   getClusterMembers()   - 
Method in class org.metaqtl. ChromCluster 
 This method returns the members of the cluster as an
 array of  Chromosomes .
   getClusters()   - 
Method in class org.metaqtl. MapCluster 
 Returns the chromosome clusters.
   getCMrkTotalNumbers()   - 
Method in class org.metaqtl. ChromCluster 
  
   getCommonMarkerPaths(ChromLayer)   - 
Method in class org.metaqtl.graph. ChromLayer 
  
   getCommonMarkerPoints(ChromLayer)   - 
Method in class org.metaqtl.graph. ChromLayer 
  
   getCopy()   - 
Method in class org.metaqtl. Qtl 
 Returns a copy of the QTL.
   getCopy()   - 
Method in class org.metaqtl. Qtl.QTLCross 
  
   getCopy()   - 
Method in class org.metaqtl. Qtl.QTLProj 
  
   getCopy()   - 
Method in class org.metaqtl. Qtl.QTLStats 
  
   getCopy()   - 
Method in class org.metaqtl. Qtl.QTLSupport 
  
   getCopy()   - 
Method in class org.metaqtl. Qtl.QTLTrait 
  
   getCritDelta(String, int[])   - 
Method in class org.metaqtl. MetaQtlResult 
  
   getCriteria()   - 
Static method in class org.metaqtl. EMCriteria 
 Returns an iterator on the criteria
   getCriteria()   - 
Method in class org.metaqtl. MetaQtlResult 
  
   getCriterion(int)   - 
Method in class org.metaqtl. EMCriteria 
 Get the criterion value according to its integer
 code.
   getCriterion(String)   - 
Method in class org.metaqtl. EMCriteria 
  
   getCriterion(int)   - 
Method in class org.metaqtl. EMResult 
  
   getCriterion()   - 
Method in class org.metaqtl. MetaQtlModel 
  
   getCriterionIdx(String)   - 
Static method in class org.metaqtl. EMCriteria 
 Returns the integer code for the criterion which matches
 the given name.
   getCritValues(String, int[])   - 
Method in class org.metaqtl. MetaQtlResult 
  
   getCritWeights(String, int[])   - 
Method in class org.metaqtl. MetaQtlResult 
  
   getCrossDesign(IBioGenome)   - 
Static method in class org.metaqtl.util. GeneticMapProperties 
 This routine returns an object which handle the
 properties of the offspring which have been used  
 to create the genetic map.
   getCrossType()   - 
Method in class org.metaqtl.bio.entity. CrossPopulation 
  
   getCrossType()   - 
Method in interface org.metaqtl.bio. IBioCross 
  
   getCSIdx(int, int)   - 
Method in class org.metaqtl. CMarkerSequence 
  
   getDataPoints(int, boolean)   - 
Method in class org.metaqtl. MetaQtlData 
 For a given trait group indice this methods returns
 the data points as a matrix of Double X[0..1][0..n-1]
 where X[0] is the vector of the n position values
 and X[1] is the vector of the n standard deviations
 of the positions.
   getDF()   - 
Method in interface org.metaqtl.numrec. NumericalUtilities.Function 
 Returns the derivative of the function.
   getDF()   - 
Method in interface org.metaqtl.numrec. NumericalUtilities.NRFunction 
 Returns the value of the derivate in x;
   getDico(String)   - 
Static method in class org.metaqtl.main. MetaMain 
  
   getDist()   - 
Method in class org.metaqtl. TreeNode 
  
   getDistance(int, int, int)   - 
Method in class org.metaqtl. Chromosome 
 This methods returns the distance between the first marker and
 the marker with the given indice using the given mapping context.
   getDistance(int)   - 
Method in class org.metaqtl. Chromosome 
 This methods returns the distance between the first marker and
 the marker with the given indice using the default mapping
 context.
   getDistanceBetween(int, int, int, int)   - 
Method in class org.metaqtl. Chromosome 
 This methods returns the distance between the markers with
 the given indices assuming that  mrkIdxL < mrkIdxR .
   getDistanceBetween(int, int)   - 
Method in class org.metaqtl. Chromosome 
  
   getDistHeterogeneity(int, int[][], Chromosome, Chromosome)   - 
Static method in class org.metaqtl. Chromosome 
  
   getDubious(String)   - 
Static method in class org.metaqtl.main. MetaMain 
  
   getEMEps()   - 
Method in class org.metaqtl.algo. QtlClustAlgorithm 
  
   getEMRanStartNumber()   - 
Method in class org.metaqtl.algo. QtlClustAlgorithm 
  
   getEntity(String)   - 
Method in class org.metaqtl.bio.entity. BioEntityContainer 
  
   getEntityBeanClass()   - 
Method in class org.metaqtl.bio.entity.factory. XmlBioEntitiesFactory 
  
   getEntityBeanClass()   - 
Method in class org.metaqtl.bio.entity.factory. XmlBioEntityFactory 
  
   getEntityBeanClass()   - 
Method in class org.metaqtl.bio.entity.factory. XmlGeneticMapFactory 
  
   getEntityBeanClass()   - 
Method in class org.metaqtl.bio.entity.factory. XmlOntologyFactory 
  
   getEntityType()   - 
Method in class org.metaqtl.bio.entity.factory. XmlBioEntitiesFactory 
  
   getEntityType()   - 
Method in class org.metaqtl.bio.entity.factory. XmlBioEntityFactory 
  
   getEntityType()   - 
Method in class org.metaqtl.bio.entity.factory. XmlGeneticMapFactory 
  
   getEntityType()   - 
Method in class org.metaqtl.bio.entity.factory. XmlOntologyFactory 
  
   getEuclidean()   - 
Method in class org.metaqtl. EMResult 
  
   getF()   - 
Method in interface org.metaqtl.numrec. NumericalUtilities.NRFunction 
 Returns the value of the function in x.
   getFirstCommonMrkIdx(Chromosome, Chromosome)   - 
Static method in class org.metaqtl. Chromosome 
  
   getFunction()   - 
Method in class org.metaqtl.bio.entity. Ontology 
  
   getFunction()   - 
Method in interface org.metaqtl.bio. IBioOntology 
 Returns the function of the ontology
   getGeneration()   - 
Method in class org.metaqtl.bio.entity. Population 
  
   getGeneration()   - 
Method in interface org.metaqtl.bio. IBioCross 
  
   getGenome()   - 
Method in class org.metaqtl.bio.entity. LocusContainer 
  
   getGenome()   - 
Method in interface org.metaqtl.bio. IBioLGroup 
  
   getGraph()   - 
Method in class org.metaqtl. ChromCluster 
  
   getGroup()   - 
Method in class org.metaqtl.bio.entity. AlleleContainer 
  
   getGroup(String)   - 
Method in class org.metaqtl.bio.entity. GroupContainer 
  
   getGroup()   - 
Method in class org.metaqtl.bio.entity. Locus 
  
   getGroup(String)   - 
Method in interface org.metaqtl.bio. IBioGenome 
  
   getGroup()   - 
Method in interface org.metaqtl.bio. IBioLocus 
  
   getGroups()   - 
Method in class org.metaqtl.bio.entity.bean. GeneticMapBean 
  
   getHeight()   - 
Method in class org.metaqtl.graph. ChromAxe 
  
   getHeight()   - 
Method in class org.metaqtl.graph. ChromLayer 
  
   getHeight()   - 
Method in class org.metaqtl.graph. GraphShape 
  
   getHeight()   - 
Method in class org.metaqtl.graph. Layer 
  
   getHeight()   - 
Method in class org.metaqtl.graph. MetaGraph 
  
   getHeight()   - 
Method in class org.metaqtl. TreeNode 
  
   getHeigth()   - 
Method in class org.metaqtl.graph. QtlShape 
  
   getID()   - 
Method in class org.metaqtl.bio.entity. OntologyTerm 
  
   getID()   - 
Method in interface org.metaqtl.bio. IBioOntologyTerm 
 Returns the id if the terms.
   getImage(ChromGraph[])   - 
Static method in class org.metaqtl.graph. GraphFactory 
  
   getIndividual(String)   - 
Method in class org.metaqtl.bio.entity. IndividualContainer 
  
   getIndividual(String)   - 
Method in interface org.metaqtl.bio. IBioPopulation 
  
   getIndividualNumber()   - 
Method in class org.metaqtl.bio.entity. IndividualContainer 
  
   getIndividualNumber()   - 
Method in class org.metaqtl.bio.entity. Population 
  
   getIndividualNumber()   - 
Method in interface org.metaqtl.bio. IBioPopulation 
  
   getInstance()   - 
Static method in class org.metaqtl.bio.util. BioEntityNameComparator 
  
   getInstance()   - 
Static method in class org.metaqtl.bio.util. LocusPositionComparator 
  
   getK()   - 
Method in class org.metaqtl. EMResult 
  
   getKeys()   - 
Method in class org.metaqtl.bio.util. TabulatedReader 
 Returns the set of the keys.
   getKValues()   - 
Method in class org.metaqtl. MetaQtlResult 
  
   getLabelSide()   - 
Method in class org.metaqtl.graph. ChromShape 
  
   getLayer(double, double)   - 
Method in class org.metaqtl.graph. QtlLayerBuffer 
 Returns a QtlLayer which represent the contents of
 the buffer.
   getLeaf(int)   - 
Method in class org.metaqtl. Tree 
  
   getLeafHeights()   - 
Method in class org.metaqtl. Tree 
  
   getLeafNumber()   - 
Method in class org.metaqtl. Tree 
  
   getLeftCSMarker(int, int)   - 
Method in class org.metaqtl. CMarkerSequence 
 Returns the indices of the marker at the left of the 
 given one.
   getLocus()   - 
Method in class org.metaqtl.bio.entity.bean. LGroupBean 
  
   getLocus(String)   - 
Method in class org.metaqtl.bio.entity. LocusContainer 
  
   getLocus()   - 
Method in interface org.metaqtl.bio. IBioAllele 
  
   getLocus(String)   - 
Method in interface org.metaqtl.bio. IBioLGroup 
  
   getLocusAdapter()   - 
Static method in class org.metaqtl.bio.entity. Locus 
  
   getLocusNumber()   - 
Method in class org.metaqtl.bio.entity. LocusContainer 
  
   getLocusNumber()   - 
Method in interface org.metaqtl.bio. IBioLGroup 
  
   getLocusType()   - 
Method in class org.metaqtl.bio.entity. Marker 
  
   getLocusType()   - 
Method in class org.metaqtl.bio.entity. QTL 
  
   getLocusType()   - 
Method in interface org.metaqtl.bio. IBioLocus 
  
   getLogger()   - 
Method in class org.metaqtl.algo. MetaAlgorithm 
 Returns the logger for this algorithm as a 
  PrintStream .
   getMahalanobis(double[])   - 
Method in class org.metaqtl. EMResult 
  
   getMap(String)   - 
Static method in class org.metaqtl.main. MetaMain 
  
   getMap(IBioGenome[], String)   - 
Static method in class org.metaqtl.main. MetaMain 
  
   getMapName()   - 
Method in class org.metaqtl. Chromosome 
  
   getMappingCross(IBioGenome)   - 
Static method in class org.metaqtl.util. GeneticMapProperties 
  
   getMappingFunction(IBioGenome)   - 
Static method in class org.metaqtl.util. GeneticMapProperties 
 This methods returns the integer code of the mapping
 function associated to the given genetic map.
   getMappingUnit(IBioGenome)   - 
Static method in class org.metaqtl.util. GeneticMapProperties 
  
   getMaps(String)   - 
Static method in class org.metaqtl.main. MetaMain 
  
   getMarkerDico()   - 
Method in class org.metaqtl. ChromCluster 
  
   getMarkerDico()   - 
Method in class org.metaqtl. MapCluster 
  
   getMarkerIdx(int, int, int)   - 
Method in class org.metaqtl. CMarkerSequence 
  
   getMarkerIdxWithName(String)   - 
Method in class org.metaqtl. Chromosome 
 This method looks if it exists a marker on the chromosome
 with the given name and returns its indice.
   getMarkerIndexByName(String)   - 
Method in class org.metaqtl. ChromCluster 
 For a given marker name this method returns
 the marker index of the marker table if found.
   getMarkerIntervalNumber()   - 
Method in class org.metaqtl. ChromCluster 
 This method returns the total number of marker intervals,
 i.e the sum over chromosomes of the number of marker intervals.
   getMarkerName(String)   - 
Method in class org.metaqtl. ChromCluster 
 This method returns the name of the locus
 from the given name.
   getMarkerName(int)   - 
Method in class org.metaqtl. Chromosome 
 Returns the names of the marker at the i^th position
 on the chromosome.
   getMarkerNameByIndex(int)   - 
Method in class org.metaqtl. ChromCluster 
 This method returns the name of the marker 
 according to this indice.
   getMarkerNumber()   - 
Method in class org.metaqtl. ChromCluster 
 This method returns the number of distinct markers implied
 in this cluster, i.e the size of the marker table.
   getMarkerOccurence(int)   - 
Method in class org.metaqtl. ChromCluster 
 Returns the number of times the marker with
 the indice i has been seen over the
 chromosomes of the cluster.
   getMetaOccurence(IBioLocus)   - 
Static method in class org.metaqtl.util. LocusProperties 
  
   getMetaQtl(String, String, int)   - 
Method in class org.metaqtl. MetaQtlAnalysis 
 Returns the meta Qtl for the given chromosome indice
  cidx , the given trait name  trait 
 and the given number of clusters  k .
   getMetaQtl(int)   - 
Method in class org.metaqtl. MetaQtlResult 
  
   getMetaQtls()   - 
Method in class org.metaqtl. Chromosome 
  
   getModel(String, String)   - 
Method in class org.metaqtl. MetaQtlModel 
  
   getModel(int, int)   - 
Method in class org.metaqtl. MetaQtlModel 
  
   getMrkTickPoint2D(int, boolean)   - 
Method in class org.metaqtl.graph. ChromLayer 
  
   getMrkTickPoint2D(int, boolean)   - 
Method in class org.metaqtl.graph. ChromShape 
  
   getMrkToRm(File)   - 
Static method in class org.metaqtl.main. MetaMain 
  
   getMu(int)   - 
Method in class org.metaqtl. EMResult 
  
   getName()   - 
Method in class org.metaqtl.bio.entity.bean. BioEntityBean 
  
   getName()   - 
Method in class org.metaqtl.bio.entity.bean. PropertyBean 
  
   getName()   - 
Method in class org.metaqtl.bio.entity. BioEntity 
  
   getName()   - 
Method in class org.metaqtl.bio.entity. BioEntityCollection 
  
   getName()   - 
Method in interface org.metaqtl.bio. IBioEntity 
 Returns the name of the biological entity
   getName()   - 
Method in class org.metaqtl. ChromCluster 
 Returns the name of the cluster.
   getName()   - 
Method in class org.metaqtl. Chromosome 
 Returns the name of the chromosome.
   getName()   - 
Method in class org.metaqtl. Qtl 
 Returns the name of the QTL.
   getName()   - 
Method in class org.metaqtl. TreeNode 
  
   getNodeHeights()   - 
Method in class org.metaqtl. Tree 
  
   getNodeNumber()   - 
Method in class org.metaqtl. Tree 
  
   getOntology(String, char)   - 
Static method in class org.metaqtl.main. MetaMain 
  
   getOption()   - 
Method in exception org.metaqtl.main. CmdLineParser.IllegalOptionValueException 
  
   getOptionChar()   - 
Method in exception org.metaqtl.main. CmdLineParser.NotFlagException 
  
   getOptionName()   - 
Method in exception org.metaqtl.main. CmdLineParser.UnknownOptionException 
  
   getOptionValue(CmdLineParser.Option)   - 
Method in class org.metaqtl.main. CmdLineParser 
 Equivalent to   getOptionValue(o,
 null)  .
   getOptionValue(CmdLineParser.Option, Object)   - 
Method in class org.metaqtl.main. CmdLineParser 
  
   getOptionValues(CmdLineParser.Option)   - 
Method in class org.metaqtl.main. CmdLineParser 
  
   getPalette(int)   - 
Static method in class org.metaqtl.graph. PaletteColor 
 Returns a palete of colors which should be distinct.
   getPaletteGradient(int, Color, Color)   - 
Static method in class org.metaqtl.graph. PaletteColor 
  
   getParent()   - 
Method in class org.metaqtl.bio.entity. BioEntity 
  
   getParent()   - 
Method in class org.metaqtl.bio.entity. BioEntityCollection 
  
   getParent()   - 
Method in interface org.metaqtl.bio. IBioEntity 
 Returns the entity that handles this entity.
   getParent()   - 
Method in class org.metaqtl. TreeNode 
  
   getParents()   - 
Method in class org.metaqtl.bio.entity. CrossPopulation 
  
   getParents()   - 
Method in class org.metaqtl.bio.entity. Individual 
  
   getParents()   - 
Method in class org.metaqtl.bio.entity. Population 
  
   getParents()   - 
Method in interface org.metaqtl.bio. IBioCross 
  
   getParents()   - 
Method in interface org.metaqtl.bio. IBioIndividual 
 Returns the pedigree of the individual
   getParentTerm()   - 
Method in class org.metaqtl.bio.entity. OntologyTerm 
  
   getParentTerm()   - 
Method in interface org.metaqtl.bio. IBioOntologyTerm 
 Returns the parent of the term.
   getPartNames()   - 
Method in class org.metaqtl. QtlPartition 
  
   getPedigree()   - 
Method in class org.metaqtl.bio.entity. Individual 
  
   getPi(int)   - 
Method in class org.metaqtl. EMResult 
  
   getPopulation()   - 
Method in class org.metaqtl.bio.entity. Individual 
  
   getPopulation()   - 
Method in interface org.metaqtl.bio. IBioIndividual 
  
   getPosition()   - 
Method in class org.metaqtl.bio.entity. AlleleContainer 
  
   getPosition()   - 
Method in class org.metaqtl.bio.entity. Locus 
  
   getPosition()   - 
Method in interface org.metaqtl.bio. IBioLocus 
  
   getProba()   - 
Method in class org.metaqtl. Chromosome 
  
   getProbaColor(double)   - 
Static method in class org.metaqtl.graph. MetaGraphPar 
  
   getProbaColor(double, double)   - 
Static method in class org.metaqtl.graph. MetaGraphPar 
  
   getProperties()   - 
Method in class org.metaqtl.bio.entity.bean. BioEntityBean 
  
   getProperties()   - 
Method in class org.metaqtl.bio.entity. BioEntity 
  
   getProperties()   - 
Method in class org.metaqtl.bio.entity. BioEntityCollection 
  
   getProperties()   - 
Method in interface org.metaqtl.bio. IBioEntity 
 Gets the properties of the entity.
   getQtl(int, int)   - 
Method in class org.metaqtl. MetaQtlData 
 Returns the j^th QTL in the i^th cluster.
   getQtlIdx(int)   - 
Method in class org.metaqtl. MetaQtlData 
 Returns the indices of the Qtl for the given
 trait cluster indice  i .
   getQTLInMrkIntProb(int, int, int)   - 
Method in class org.metaqtl. Chromosome 
 This methods returns the probability that the QTL be inside 
 the marker interval with the given right and left marker indices
 and the given QTL indice.
   getQTLInMrkIntProb(int, int, Qtl)   - 
Method in class org.metaqtl. Chromosome 
 This methods returns the probability that the QTL be inside 
 the marker interval with the given right and left marker indices
 and the given QTL.
   getQtlNumber()   - 
Method in class org.metaqtl. Chromosome 
  
   getQtlNumber()   - 
Method in class org.metaqtl. MetaQtlData 
  
   getQtlPartition(Chromosome, IBioOntology)   - 
Static method in class org.metaqtl.main. MetaMain 
  
   getQtlPartition(Chromosome, MetaQtlAnalysis, String, int, char)   - 
Static method in class org.metaqtl.main. MetaMain 
  
   getQtlPartNames()   - 
Method in class org.metaqtl.graph. QtlPartitionLayer 
  
   getQtls()   - 
Method in class org.metaqtl. Chromosome 
 This method returns a copy of the QTL located
 on thic chromosome.
   getQTLSD(Qtl.QTLSupport, int, int)   - 
Static method in class org.metaqtl. Qtl 
 Depending on the CI type this methods returns the  
 the standard deviation of the QTL position using the given
 mapping context.
   getQTLSD(double, Qtl.QTLCross, int)   - 
Static method in class org.metaqtl. Qtl 
 Depending on the r-square parameter and the cross design
 this method returns the standard deviation of the QTL position
 for the given mapping context.
   getQtlTree()   - 
Method in class org.metaqtl. MetaQtlResult 
  
   getRecHeterogeneity(int, int[][], Chromosome, Chromosome)   - 
Static method in class org.metaqtl. Chromosome 
  
   getRemainingArgs()   - 
Method in class org.metaqtl.main. CmdLineParser 
  
   getResult()   - 
Method in class org.metaqtl.algo. ClustAlgorithm 
 When the algorithm is finished use this method to get
 the result.
   getResult()   - 
Method in class org.metaqtl.algo. MetaMapAlgorithm 
  
   getResult()   - 
Method in class org.metaqtl.algo. QtlProjAlgorithm 
  
   getResult(String)   - 
Static method in class org.metaqtl.main. MetaMain 
  
   getResult(String, String)   - 
Method in class org.metaqtl. MetaQtlAnalysis 
 Returns the result for the given trait name or null
 if not found.
   getResult(String)   - 
Method in class org.metaqtl. MetaQtlAnalysis 
 Returns the first result for the given chromosome.
   getResult(int, String)   - 
Method in class org.metaqtl. MetaQtlAnalysis 
 Returns the result for the given trait name or null
 if not found.
   getResults(String)   - 
Method in class org.metaqtl. MetaQtlAnalysis 
  
   getRightCSMarker(int, int)   - 
Method in class org.metaqtl. CMarkerSequence 
  
   getRoot()   - 
Method in class org.metaqtl.bio.entity. Ontology 
  
   getRoot()   - 
Method in interface org.metaqtl.bio. IBioOntology 
 Returns the root of the ontology tree.
   getRoot()   - 
Method in class org.metaqtl. Tree 
  
   getSD(int)   - 
Method in class org.metaqtl. EMResult 
  
   getSize()   - 
Method in class org.metaqtl.bio.entity. Population 
 Returns the size of the population.
   getSize()   - 
Method in interface org.metaqtl.bio. IBioCross 
  
   getSubChromosome(String[])   - 
Method in class org.metaqtl. Chromosome 
  
   getSuboption()   - 
Method in exception org.metaqtl.main. CmdLineParser.UnknownSuboptionException 
  
   getSynonymous(String)   - 
Method in class org.metaqtl. MetaDico 
  
   getTerm(IBioOntologyTerm)   - 
Method in class org.metaqtl.bio.entity. Ontology 
  
   getTerm(String)   - 
Method in class org.metaqtl.bio.entity. Ontology 
  
   getTerm(IBioOntologyTerm)   - 
Method in interface org.metaqtl.bio. IBioOntology 
 Returns if exists the term in the ontology which 
 matches the given one.
   getTerm(String)   - 
Method in interface org.metaqtl.bio. IBioOntology 
 This method looks into the ontology if there is 
 a term with the given name and returns it.
   getTerm(String)   - 
Method in class org.metaqtl. MetaDico 
  
   getTerms()   - 
Method in class org.metaqtl. MetaDico 
  
   getToken()   - 
Method in class org.metaqtl.bio.util. TabulatedReader 
  
   getToken()   - 
Method in class org.metaqtl.bio.util. TabulatedWriter 
  
   getTraitClusterName(int)   - 
Method in class org.metaqtl. MetaQtlData 
  
   getTraitClusterNames()   - 
Method in class org.metaqtl. MetaQtlData 
 Returns the names of the trait clusters.
   getTraitClusterNumber()   - 
Method in class org.metaqtl. MetaQtlData 
 Returns the number of trait groups for this data set as
 defined by the clustering method.
   getTraitClusterSize(int)   - 
Method in class org.metaqtl. MetaQtlData 
 Returns the size of the trait cluster i.
   getTraitIdx(int, String)   - 
Method in class org.metaqtl. MetaQtlAnalysis 
  
   getTraitName()   - 
Method in class org.metaqtl. MetaQtlResult 
 Returns the name of the trait cluster for this result.
   getTraitNames(String)   - 
Method in class org.metaqtl. MetaQtlModel 
  
   getTraitNames(int)   - 
Method in class org.metaqtl. MetaQtlModel 
  
   getType()   - 
Method in class org.metaqtl.bio.entity. AlleleContainer 
  
   getType()   - 
Method in class org.metaqtl.bio.entity. BioEntity 
  
   getType()   - 
Method in class org.metaqtl.bio.entity. BioEntityCollection 
  
   getType()   - 
Method in class org.metaqtl.bio.entity. CrossPopulation 
  
   getType()   - 
Method in class org.metaqtl.bio.entity. GeneticMap 
  
   getType()   - 
Method in class org.metaqtl.bio.entity. GroupContainer 
  
   getType()   - 
Method in class org.metaqtl.bio.entity. Individual 
  
   getType()   - 
Method in class org.metaqtl.bio.entity. IndividualContainer 
  
   getType()   - 
Method in class org.metaqtl.bio.entity. LGroup 
  
   getType()   - 
Method in class org.metaqtl.bio.entity. LocusContainer 
  
   getType()   - 
Method in class org.metaqtl.bio.entity. Marker 
  
   getType()   - 
Method in class org.metaqtl.bio.entity. Ontology 
  
   getType()   - 
Method in class org.metaqtl.bio.entity. OntologyTerm 
  
   getType()   - 
Method in class org.metaqtl.bio.entity. Population 
  
   getType()   - 
Method in class org.metaqtl.bio.entity. QTL 
  
   getType()   - 
Method in interface org.metaqtl.bio. IBioEntity 
 There are 2 main class of entities.
   getType()   - 
Method in interface org.metaqtl.bio. IBioLocus 
  
   getType()   - 
Method in class org.metaqtl.bio.util. EstimatedLocusPosition 
  
   getType()   - 
Method in interface org.metaqtl.bio.util. ILGroupPosition 
  
   getType()   - 
Method in class org.metaqtl.bio.util. LGroupPosition 
  
   getType()   - 
Method in class org.metaqtl.bio.util. StaticLocusPosition 
  
   getUSD(EMResult, int, int, String)   - 
Method in class org.metaqtl. MetaQtlResult 
  
   getValue()   - 
Method in class org.metaqtl.bio.entity.bean. PropertyBean 
  
   getValue()   - 
Method in exception org.metaqtl.main. CmdLineParser.IllegalOptionValueException 
  
   getValue(String, Locale)   - 
Method in class org.metaqtl.main. CmdLineParser.Option 
  
   getVLabels()   - 
Method in class org.metaqtl. ChromCluster.ClusterGraph 
  
   getWidth()   - 
Method in class org.metaqtl.graph. ChromLayer 
  
   getWidth()   - 
Method in class org.metaqtl.graph. GraphShape 
  
   getWidth()   - 
Method in class org.metaqtl.graph. Layer 
  
   getWidth()   - 
Method in class org.metaqtl.graph. MetaGraph 
  
   getWidth()   - 
Method in class org.metaqtl.graph. QtlShape 
  
   getWorkAmount()   - 
Method in class org.metaqtl.algo. MetaAlgorithm 
  
   getWorkAmount()   - 
Method in interface org.metaqtl. IMetaAlgorithm 
 Returns the amount of the work to do.
   getWorkProgress()   - 
Method in class org.metaqtl.algo. MetaAlgorithm 
  
   getWorkProgress()   - 
Method in interface org.metaqtl. IMetaAlgorithm 
 Returns the amount of work which have already
 been done.
   getX()   - 
Method in class org.metaqtl.graph. AnchoredElement 
  
   getX()   - 
Method in class org.metaqtl.graph. ChromAxe 
  
   getX()   - 
Method in class org.metaqtl. TreeNode 
  
   getXBestPred()   - 
Method in class org.metaqtl. EMResult 
  
   getXChrom()   - 
Method in class org.metaqtl.graph. ChromShape 
  
   getXPred()   - 
Method in class org.metaqtl. EMResult 
  
   getXPredIdx(int)   - 
Method in class org.metaqtl. EMResult 
  
   getY()   - 
Method in class org.metaqtl.graph. AnchoredElement 
  
   getY()   - 
Method in class org.metaqtl. TreeNode 
  
   getYDistance(QtlShape, QtlShape)   - 
Static method in class org.metaqtl.graph. QtlShape 
 Computes the distance between two Qtl shapes assuming
 along the same axe Y (i.e the shapes have same X).
   getYMax()   - 
Method in class org.metaqtl.graph. ChromAxe 
  
   getYMax()   - 
Method in class org.metaqtl.graph. ChromLayer 
  
   getYMax()   - 
Method in class org.metaqtl.graph. ChromShape 
  
   getYMax()   - 
Method in class org.metaqtl.graph. GraphShape 
  
   getYMax()   - 
Method in class org.metaqtl.graph. Layer 
  
   getYMax()   - 
Method in class org.metaqtl.graph. QtlLayer 
  
   getYMax()   - 
Method in class org.metaqtl.graph. QtlPartitionLayer 
  
   getYMax()   - 
Method in class org.metaqtl.graph. QtlShape 
  
   getYMax()   - 
Method in class org.metaqtl.graph. QtlTreeLayer 
  
   getYMin()   - 
Method in class org.metaqtl.graph. ChromAxe 
  
   getYMin()   - 
Method in class org.metaqtl.graph. ChromLayer 
  
   getYMin()   - 
Method in class org.metaqtl.graph. ChromShape 
  
   getYMin()   - 
Method in class org.metaqtl.graph. GraphShape 
  
   getYMin()   - 
Method in class org.metaqtl.graph. Layer 
  
   getYMin()   - 
Method in class org.metaqtl.graph. QtlLayer 
  
   getYMin()   - 
Method in class org.metaqtl.graph. QtlPartitionLayer 
  
   getYMin()   - 
Method in class org.metaqtl.graph. QtlShape 
  
   getYMin()   - 
Method in class org.metaqtl.graph. QtlTreeLayer 
  
   getZ(int)   - 
Method in class org.metaqtl. EMResult 
  
   GraphFactory   - Class in  org.metaqtl.graph      GraphFactory()   - 
Constructor for class org.metaqtl.graph. GraphFactory 
  
   GraphShape   - Class in  org.metaqtl.graph      GraphShape()   - 
Constructor for class org.metaqtl.graph. GraphShape 
  
   group   - 
Variable in class org.metaqtl. Qtl.QTLTrait 
 The name of the group to which
 the trait belongs.
   GroupContainer   - Class in  org.metaqtl.bio.entity  Class Description Here   GroupContainer()   - 
Constructor for class org.metaqtl.bio.entity. GroupContainer 
  
   GroupContainer(String, IBioEntity)   - 
Constructor for class org.metaqtl.bio.entity. GroupContainer 
  
   groupNumber()   - 
Method in class org.metaqtl.bio.entity. GroupContainer 
  
   groupNumber()   - 
Method in interface org.metaqtl.bio. IBioGenome 
  
   groups   - 
Variable in class org.metaqtl.bio.entity.bean. GeneticMapBean 
  
   groups()   - 
Method in class org.metaqtl.bio.entity. GroupContainer 
  
   groups()   - 
Method in interface org.metaqtl.bio. IBioGenome 
  
   gser(double, double)   - 
Static method in class org.metaqtl.numrec. NumericalUtilities 
  
 
 
    
 H  
 
   hasChildren()   - 
Method in class org.metaqtl.bio.entity. OntologyTerm 
  
   hasChildren()   - 
Method in interface org.metaqtl.bio. IBioOntologyTerm 
 Returns true if the term has children, false
 otherwise.
   hasNext()   - 
Method in class org.metaqtl.bio.util. TabulatedReader 
 Returns true if there is still a line to read.
   hasProba()   - 
Method in class org.metaqtl.graph. ChromLayer 
  
   hasQTL()   - 
Method in class org.metaqtl. Chromosome 
 This method tests if the chromosome have a 
 set of QTL defined.
   haveSameFrame(int, int)   - 
Method in class org.metaqtl. CMarkerSequence 
  
   HClustAlgorithm   - Class in  org.metaqtl.algo  This class implements stantard agglomerative hierarchical
 clustering methods.   HClustAlgorithm()   - 
Constructor for class org.metaqtl.algo. HClustAlgorithm 
  
   HEADER   - 
Static variable in class org.metaqtl.bio.entity.factory. OntologyTabFactory 
  
   HEIGHT   - 
Static variable in class org.metaqtl.graph. GraphFactory 
  
   height   - 
Variable in class org.metaqtl.graph. GraphShape 
  
   height   - 
Variable in class org.metaqtl.graph. Layer 
 The height of the layer.
   height   - 
Variable in class org.metaqtl. TreeNode 
 The distance between the node and the root of the tree (if defined).
   help   - 
Variable in class org.metaqtl.main. MetaMain 
  
   HORIZONTAL_MODE   - 
Static variable in class org.metaqtl. QtlPartition 
  
 
 
    
 I  
 
   IBioAdaptable   - Interface in  org.metaqtl.bio      IBioAdapter   - Interface in  org.metaqtl.bio      IBioAllele   - Interface in  org.metaqtl.bio  Class Description Here   IBioConstants   - Interface in  org.metaqtl.bio.entity  Class Description Here   IBioCross   - Interface in  org.metaqtl.bio  Class Description Here   IBioCrossTypes   - Interface in  org.metaqtl.bio  Class Description Here   IBioEntity   - Interface in  org.metaqtl.bio  Class Description Here   IBioEntityTypes   - Interface in  org.metaqtl.bio  Class Description Here   IBioGenome   - Interface in  org.metaqtl.bio      IBioGenomeProperties   - Interface in  org.metaqtl  This interface defines both the keys and the 
 default values of properties which
 can be handled by a org.thalia.bio.entity.IBioGenome.   IBioIndividual   - Interface in  org.metaqtl.bio  Class Description Here   IBioIndividualTree   - Interface in  org.metaqtl.bio  Class Description Here   IBioLGroup   - Interface in  org.metaqtl.bio      IBioLGroupProperties   - Interface in  org.metaqtl  This interface defines the key of properties which can
 be handled by a org.thalia.bio.entity.IBioLGroup.   IBioLocus   - Interface in  org.metaqtl.bio      IBioLocusProperties   - Interface in  org.metaqtl  This interface defines the key of the properties which
 can be handled by a org.thalia.bio.entity.IBioLocus.   IBioOntology   - Interface in  org.metaqtl.bio  This interface defines some rules to handle a ontology.   IBioOntologyTerm   - Interface in  org.metaqtl.bio  This class defines a term of an ontology.   IBioPopulation   - Interface in  org.metaqtl.bio  Class Description Here   ic1   - 
Variable in class org.metaqtl. Qtl.QTLSupport 
 The recombination rate between the QTL position
 and the left border of the IC.
   ic2   - 
Variable in class org.metaqtl. Qtl.QTLSupport 
 The recombination rate between the QTL position
 and the right border of the IC.
   ICOMP   - 
Static variable in class org.metaqtl. EMCriteria 
  
   icomp   - 
Variable in class org.metaqtl. EMCriteria 
  
   ICOMP_NAME   - 
Static variable in class org.metaqtl. EMCriteria 
  
   id   - 
Variable in class org.metaqtl.bio.entity.bean. OntologyTermBean 
 The id of the term.
   id   - 
Variable in class org.metaqtl. Qtl 
 The id of the Qtl
   idx   - 
Variable in class org.metaqtl. TreeNode 
 ?
 The identifiant of the cluster.
   idx1   - 
Variable in class org.metaqtl. CMarkerSequence 
 The indices of the markers on the first
 chromosome for the common sequences.
   idx2   - 
Variable in class org.metaqtl. CMarkerSequence 
 The indices of the marker on the second
 chromosome for the common sequences.
   IGeneticMapProperties   - Interface in  org.metaqtl.bio.entity  Class Description Here   ILGroupPosition   - Interface in  org.metaqtl.bio.util  Class Description Here   IMetaAlgorithm   - Interface in  org.metaqtl  This interface defines some methods which are common
 to data analysis algorithms.   IMetaQtlConstants   - Interface in  org.metaqtl  This interface defines some global constants which are used
 by several classes of the package.   IMIN(int, int)   - 
Static method in class org.metaqtl.numrec. NumericalUtilities 
 Returns the min between  a  and  b 
   incms   - 
Variable in class org.metaqtl. CMarkerSequence 
  
   indexx(int, double[], int[])   - 
Static method in class org.metaqtl.numrec. NumericalUtilities 
 Sort the given array of double arrin[1..n] without destroying it and
 returns the sorted indices of the array elements into indx[1..n].
   Individual   - Class in  org.metaqtl.bio.entity  Class Description Here   Individual()   - 
Constructor for class org.metaqtl.bio.entity. Individual 
  
   Individual(String, IBioEntity)   - 
Constructor for class org.metaqtl.bio.entity. Individual 
  
   INDIVIDUAL   - 
Static variable in interface org.metaqtl.bio. IBioEntityTypes 
 The integer code for an individual entity
   IndividualContainer   - Class in  org.metaqtl.bio.entity  Class Description Here   IndividualContainer()   - 
Constructor for class org.metaqtl.bio.entity. IndividualContainer 
  
   IndividualContainer(String, IBioEntity)   - 
Constructor for class org.metaqtl.bio.entity. IndividualContainer 
  
   individuals()   - 
Method in class org.metaqtl.bio.entity. IndividualContainer 
  
   individuals()   - 
Method in interface org.metaqtl.bio. IBioPopulation 
  
   InfoMap   - Class in  org.metaqtl.main      InfoMap()   - 
Constructor for class org.metaqtl.main. InfoMap 
  
   init(Graphics2D, ChromGraph[])   - 
Method in class org.metaqtl.graph. MetaGraph 
  
   initCmdLineParser()   - 
Method in class org.metaqtl.main. MetaMain 
  
   initEM(double[], double[], EMResult, Random)   - 
Static method in class org.metaqtl.algo. EMAlgorithm 
 Initializes the EM algorithm by randomly assigning observations to
 the clusters and do one M-Step to compute the first values of the 
 parameters.
   initNodeIdx()   - 
Method in class org.metaqtl. Tree 
  
   initPalettes()   - 
Static method in class org.metaqtl.graph. MetaGraphPar 
  
   initProbaPalette()   - 
Static method in class org.metaqtl.graph. MetaGraphPar 
  
   initQTLPalette()   - 
Static method in class org.metaqtl.graph. MetaGraphPar 
  
   initQTLPalette(int)   - 
Static method in class org.metaqtl.graph. MetaGraphPar 
  
   initReverse()   - 
Method in class org.metaqtl. Tree 
  
   intScale   - 
Variable in class org.metaqtl. Qtl.QTLProj 
 The scale ratio between the original flanking
 marker interval and the one on the map used
 for projection.
   IOntologyTermProperties   - Interface in  org.metaqtl.bio      IRISe   - 
Static variable in interface org.metaqtl.bio. IBioCrossTypes 
  
   IRISi   - 
Static variable in interface org.metaqtl.bio. IBioCrossTypes 
  
   isConnected(int)   - 
Method in class org.metaqtl. ChromCluster.ClusterGraph 
 Test if the graph is connected
   isConnected(int)   - 
Method in class org.metaqtl. ChromCluster 
 Tests if the cluster of chromosome is connected using a graph
 approach.
   isDubiousLocus(IBioLocus)   - 
Method in class org.metaqtl. ChromCluster 
 If a list of dubious markers has been set before
 then this routine checks if the given locus is
 not included into this list.
   isDubiousOrderLocus(IBioLocus)   - 
Static method in class org.metaqtl.util. LocusProperties 
 Returns true if the given locus has a property order.dubious
 set to one, false otherwise
   isIgnore()   - 
Method in class org.metaqtl. Qtl 
 This methods returns true if the QTL must be ignored.
   isLoggerEnable()   - 
Method in class org.metaqtl.algo. MetaAlgorithm 
  
   ISWAP(int[], int, int)   - 
Static method in class org.metaqtl.numrec. NumericalUtilities 
 Swaps elements i and j in a.
   iterate(double[], double[], EMResult)   - 
Static method in class org.metaqtl.algo. EMAlgorithm 
 This method performs one iteration of the EM-algorithm.
 
 
    
 K  
 
   k   - 
Variable in class org.metaqtl. EMResult 
 The number of clusters.
 
 
    
 L  
 
   label   - 
Variable in class org.metaqtl. TreeNode 
 The label of the cluster
   Layer   - Class in  org.metaqtl.graph      Layer(double, double)   - 
Constructor for class org.metaqtl.graph. Layer 
 Creates a layer without any width and heigth
 constraints which upper left corner will be
 located at the point (x,y).
   LAYER_HSPACE   - 
Static variable in class org.metaqtl.graph. MetaGraphPar 
 The horizontal space between layers.
   LAYER_VSPACE   - 
Static variable in class org.metaqtl.graph. MetaGraphPar 
 The vertical space between layers.
   leaf   - 
Variable in class org.metaqtl. TreeNode 
 The status of the node.
   LEFT_LABEL   - 
Static variable in class org.metaqtl.graph. ChromLayer 
  
   LEFT_LABEL   - 
Static variable in class org.metaqtl.graph. ChromShape 
  
   LEGEND_BOX_CEX   - 
Static variable in class org.metaqtl.graph. MetaGraphPar 
  
   LEGEND_FONT   - 
Static variable in class org.metaqtl.graph. MetaGraphPar 
  
   LEGEND_GRAD_HEIGHT   - 
Static variable in class org.metaqtl.graph. MetaGraphPar 
  
   LEGEND_GRAD_WIDTH   - 
Static variable in class org.metaqtl.graph. MetaGraphPar 
  
   LEGEND_HSPACE   - 
Static variable in class org.metaqtl.graph. MetaGraphPar 
  
   LEGEND_PART_HEIGHT   - 
Static variable in class org.metaqtl.graph. MetaGraphPar 
  
   LEGEND_PART_WIDTH   - 
Static variable in class org.metaqtl.graph. MetaGraphPar 
  
   LEGEND_SCALE_UNIT   - 
Static variable in class org.metaqtl.graph. MetaGraphPar 
  
   legendLayer   - 
Variable in class org.metaqtl.graph. MetaGraph 
  
   LGroup   - Class in  org.metaqtl.bio.entity  Class Description Here   LGroup()   - 
Constructor for class org.metaqtl.bio.entity. LGroup 
  
   LGroup(String, IBioEntity)   - 
Constructor for class org.metaqtl.bio.entity. LGroup 
  
   LGROUP   - 
Static variable in interface org.metaqtl.bio. IBioEntityTypes 
 The integer code for Linkage Group entity
   LGroupBean   - Class in  org.metaqtl.bio.entity.bean  Class Description Here   LGroupBean()   - 
Constructor for class org.metaqtl.bio.entity.bean. LGroupBean 
  
   LGroupBeanAdapter   - Class in  org.metaqtl.bio.entity.adapter  Class Description Here   LGroupBeanAdapter()   - 
Constructor for class org.metaqtl.bio.entity.adapter. LGroupBeanAdapter 
  
   LGroupPosition   - Class in  org.metaqtl.bio.util  Class Description Here   LGroupPosition(double)   - 
Constructor for class org.metaqtl.bio.util. LGroupPosition 
  
   LGroupPosition(double, double)   - 
Constructor for class org.metaqtl.bio.util. LGroupPosition 
  
   load(Reader)   - 
Method in class org.metaqtl.bio.entity.factory. BioEntityFactory 
  
   load(InputStream)   - 
Method in class org.metaqtl.bio.entity.factory. BioEntityFactory 
  
   load(Reader)   - 
Method in class org.metaqtl.bio.entity.factory. MapChartFactory 
 TODO (non-Javadoc)
   load(InputStream)   - 
Method in class org.metaqtl.bio.entity.factory. MapChartFactory 
 TODO (non-Javadoc)
   load(Reader)   - 
Method in class org.metaqtl.bio.entity.factory. MapMakerFactory 
  
   load(InputStream)   - 
Method in class org.metaqtl.bio.entity.factory. MapMakerFactory 
  
   load(InputStream)   - 
Method in class org.metaqtl.bio.entity.factory. MapRawFactory 
  
   load(Reader)   - 
Method in class org.metaqtl.bio.entity.factory. MapRawFactory 
  
   load(Reader)   - 
Method in class org.metaqtl.bio.entity.factory. MapTabFactory 
  
   load(InputStream)   - 
Method in class org.metaqtl.bio.entity.factory. MapTabFactory 
  
   load(InputStream)   - 
Method in class org.metaqtl.bio.entity.factory. OntologyTabFactory 
  
   load(Reader)   - 
Method in class org.metaqtl.bio.entity.factory. OntologyTabFactory 
 
         Term_id        : the term identifient.
   load(InputStream)   - 
Method in class org.metaqtl.bio.entity.factory. XmlBioEntityFactory 
  
   load(Reader)   - 
Method in class org.metaqtl.bio.entity.factory. XmlBioEntityFactory 
  
   loci()   - 
Method in class org.metaqtl.bio.entity. LocusContainer 
  
   loci()   - 
Method in interface org.metaqtl.bio. IBioLGroup 
  
   locus   - 
Variable in class org.metaqtl.bio.entity.bean. LGroupBean 
  
   Locus   - Class in  org.metaqtl.bio.entity      Locus()   - 
Constructor for class org.metaqtl.bio.entity. Locus 
  
   Locus(String, IBioEntity)   - 
Constructor for class org.metaqtl.bio.entity. Locus 
  
   LOCUS   - 
Static variable in interface org.metaqtl.bio. IBioEntityTypes 
 The integer code for Locus entity
   LocusBean   - Class in  org.metaqtl.bio.entity.bean  Class Description Here   LocusBean()   - 
Constructor for class org.metaqtl.bio.entity.bean. LocusBean 
  
   LocusBeanAdapter   - Class in  org.metaqtl.bio.entity.adapter  Class Description Here   LocusBeanAdapter()   - 
Constructor for class org.metaqtl.bio.entity.adapter. LocusBeanAdapter 
  
   LocusContainer   - Class in  org.metaqtl.bio.entity  Class Description Here   LocusContainer()   - 
Constructor for class org.metaqtl.bio.entity. LocusContainer 
  
   LocusContainer(String, IBioEntity)   - 
Constructor for class org.metaqtl.bio.entity. LocusContainer 
  
   locusNames   - 
Variable in class org.metaqtl. ChromCluster 
 Ths locus name index table
   LocusPositionComparator   - Class in  org.metaqtl.bio.util  Class Description Here   LocusProperties   - Class in  org.metaqtl.util      LocusProperties()   - 
Constructor for class org.metaqtl.util. LocusProperties 
  
   lod   - 
Variable in class org.metaqtl. Qtl.QTLStats 
 The lod score of the QTL.
   lodDecrease   - 
Variable in class org.metaqtl. Qtl.QTLSupport 
 The value of the LOD decrease used to obtain the CI.
   logger   - 
Variable in class org.metaqtl.algo. MetaAlgorithm 
 The logger.
   loggerUp   - 
Variable in class org.metaqtl.algo. MetaAlgorithm 
 The logger status.
   longForm()   - 
Method in class org.metaqtl.main. CmdLineParser.Option 
  
 
 
    
 M  
 
   m   - 
Variable in class org.metaqtl. MultiFitResult 
 The number of parameters
   m1   - 
Variable in class org.metaqtl. Qtl 
 The indice of the left flanking marker.
   m2   - 
Variable in class org.metaqtl. Qtl 
 The indice of the right flanking marker.
   main(String[])   - 
Static method in class org.metaqtl.main. A2Xml 
  
   main(String[])   - 
Static method in class org.metaqtl.main. ConsMap 
  
   main(String[])   - 
Static method in class org.metaqtl.main. InfoMap 
  
   main(String[])   - 
Static method in class org.metaqtl.main. MapView 
  
   main(String[])   - 
Static method in class org.metaqtl.main. MetaDB 
  
   main(String[])   - 
Static method in class org.metaqtl.main. MMapView 
  
   main(String[])   - 
Static method in class org.metaqtl.main. MQTLView 
  
   main(String[])   - 
Static method in class org.metaqtl.main. QTLClust 
  
   main(String[])   - 
Static method in class org.metaqtl.main. QTLClustInfo 
  
   main(String[])   - 
Static method in class org.metaqtl.main. QTLModel 
  
   main(String[])   - 
Static method in class org.metaqtl.main. QTLProj 
  
   main(String[])   - 
Static method in class org.metaqtl.main. QTLTree 
  
   main(String[])   - 
Static method in class org.metaqtl.main. Xml2A 
  
   MAKER_NAME_VSPACE   - 
Static variable in class org.metaqtl.graph. MetaGraphPar 
  
   manageMissingData(int)   - 
Method in class org.metaqtl. MetaQtlData 
 Manage the missing data.
   MapChartFactory   - Class in  org.metaqtl.bio.entity.factory  General comments must be added here.   MapChartFactory()   - 
Constructor for class org.metaqtl.bio.entity.factory. MapChartFactory 
  
   MapCluster   - Class in  org.metaqtl      MapCluster()   - 
Constructor for class org.metaqtl. MapCluster 
 Creates a new instance of  MapCluster .
   MapDBFactory   - Class in  org.metaqtl.factory  A factory to read the list and the properties of a set
 of genetic maps.   MapDBFactory()   - 
Constructor for class org.metaqtl.factory. MapDBFactory 
  
   MapMakerFactory   - Class in  org.metaqtl.bio.entity.factory      MapMakerFactory()   - 
Constructor for class org.metaqtl.bio.entity.factory. MapMakerFactory 
  
   mapName   - 
Variable in class org.metaqtl. Chromosome 
 The name of the map in which this chromosome
 is defined.
   mapNames   - 
Variable in class org.metaqtl. MetaChrom 
 The names of the genetic maps.
   mapOrigin   - 
Variable in class org.metaqtl. Qtl.QTLProj 
 The name of the map from which
 this QTL comes.
   MAPPING_CROSS   - 
Static variable in interface org.metaqtl. IBioGenomeProperties 
 The key for mapping cross.
   MAPPING_EXPANSION   - 
Static variable in interface org.metaqtl. IBioGenomeProperties 
 The key for mapping expansion status.
   MAPPING_FUNCTION   - 
Static variable in interface org.metaqtl. IBioGenomeProperties 
 The key for mapping function.
   MAPPING_FUNCTION_HALDANE   - 
Static variable in interface org.metaqtl. IBioGenomeProperties 
 The code for Haldane mapping function.
   MAPPING_FUNCTION_HALDANE   - 
Static variable in interface org.metaqtl. IMetaQtlConstants 
 Integer code for haldane mapping function
   MAPPING_FUNCTION_KOSAMBI   - 
Static variable in interface org.metaqtl. IBioGenomeProperties 
 The code for Kosambi mapping function.
   MAPPING_FUNCTION_KOSAMBI   - 
Static variable in interface org.metaqtl. IMetaQtlConstants 
 Integer code for kosambi mapping function
   MAPPING_FUNCTION_UNKNOWN   - 
Static variable in interface org.metaqtl. IMetaQtlConstants 
 Integer code for not defined mapping function
   MAPPING_UNIT   - 
Static variable in interface org.metaqtl. IBioGenomeProperties 
 The mapping unit key.
   MAPPING_UNIT_CM   - 
Static variable in interface org.metaqtl. IBioGenomeProperties 
 The code for centi-Morgan unit.
   MAPPING_UNIT_CM   - 
Static variable in interface org.metaqtl. IMetaQtlConstants 
 Integer code for centi morgan mapping unit
   MAPPING_UNIT_M   - 
Static variable in interface org.metaqtl. IBioGenomeProperties 
 The code for Morgan unit.
   MAPPING_UNIT_M   - 
Static variable in interface org.metaqtl. IMetaQtlConstants 
 Integer code for morgan mapping unit
   MAPPING_UNIT_UNKNOWN   - 
Static variable in interface org.metaqtl. IMetaQtlConstants 
 Integer code for unknown mapping unit
   MappingFunction   - Class in  org.metaqtl.util  define all cartogarphies functions   MappingFunction()   - 
Constructor for class org.metaqtl.util. MappingFunction 
  
   MapRawFactory   - Class in  org.metaqtl.bio.entity.factory      MapRawFactory()   - 
Constructor for class org.metaqtl.bio.entity.factory. MapRawFactory 
  
   mapScale   - 
Variable in class org.metaqtl. Qtl.QTLProj 
 The scale ratio between the original
 map and the map on which the QTL has
 been projected.
   MapTabFactory   - Class in  org.metaqtl.bio.entity.factory      MapTabFactory()   - 
Constructor for class org.metaqtl.bio.entity.factory. MapTabFactory 
  
   MapView   - Class in  org.metaqtl.main      MapView()   - 
Constructor for class org.metaqtl.main. MapView 
  
   mapWeight   - 
Variable in class org.metaqtl. Qtl.QTLProj 
 The cumulated scale weights of the common
 marke sequences between the original map
 and the map on which the QTL has been projected.
   Marker   - Class in  org.metaqtl.bio.entity  Class Description Here   Marker()   - 
Constructor for class org.metaqtl.bio.entity. Marker 
  
   Marker(String, IBioEntity)   - 
Constructor for class org.metaqtl.bio.entity. Marker 
  
   MARKER   - 
Static variable in interface org.metaqtl.bio. IBioLocus 
  
   MARKER_NAME_FONT   - 
Static variable in class org.metaqtl.graph. MetaGraphPar 
  
   MARKER_POSITION_FONT   - 
Static variable in class org.metaqtl.graph. MetaGraphPar 
  
   MARKERLOCUS   - 
Static variable in interface org.metaqtl.bio. IBioEntityTypes 
  
   mcidx   - 
Variable in class org.metaqtl. CMarkerSequence 
 The common marker indices on the chromosomes.
   merge(GeneticMap)   - 
Method in class org.metaqtl.bio.entity. GeneticMap 
  Deprecated.     
   mergeGenome(IBioGenome, IBioGenome)   - 
Static method in class org.metaqtl.bio.util. BioUtilities 
 This method merges two genomes in a single one :  g1  is
 modified and will contain the result of the merge.
   MetaAlgorithm   - Class in  org.metaqtl.algo      MetaAlgorithm()   - 
Constructor for class org.metaqtl.algo. MetaAlgorithm 
  
   MetaChrom   - Class in  org.metaqtl  A MetaChrom is the result of the merge of the members of a
   ChromCluster  .   MetaChrom(MultiFitResult, ChromCluster)   - 
Constructor for class org.metaqtl. MetaChrom 
 Get a new instance of  MetaChrom  which is 
 initialized by using the result
 of the merge of the members of a   ChromCluster  .
   MetaDB   - Class in  org.metaqtl.main      MetaDB()   - 
Constructor for class org.metaqtl.main. MetaDB 
  
   MetaDico   - Class in  org.metaqtl  This class represents a dictionnary of synonyms.   MetaDico()   - 
Constructor for class org.metaqtl. MetaDico 
  
   MetaDicoFactory   - Class in  org.metaqtl.factory      MetaDicoFactory()   - 
Constructor for class org.metaqtl.factory. MetaDicoFactory 
  
   MetaGraph   - Class in  org.metaqtl.graph      MetaGraph()   - 
Constructor for class org.metaqtl.graph. MetaGraph 
  
   MetaGraphPar   - Class in  org.metaqtl.graph      MetaGraphParFactory   - Class in  org.metaqtl.factory      MetaGraphParFactory()   - 
Constructor for class org.metaqtl.factory. MetaGraphParFactory 
  
   MetaMain   - Class in  org.metaqtl.main      MetaMain()   - 
Constructor for class org.metaqtl.main. MetaMain 
  
   MetaMap   - Class in  org.metaqtl  A  MetaMap  is a bundle of   MetaChrom   objects.   MetaMap(int)   - 
Constructor for class org.metaqtl. MetaMap 
 Creates a new instance of  MetaMap 
 which will contain  nchr    MetaChrom  
 objects.
   MetaMapAdapter   - Class in  org.metaqtl.adapter  This class defines some usefull methods to adapt   MetaMap  
 objects into other kinds of objects or the inverse.   MetaMapAdapter()   - 
Constructor for class org.metaqtl.adapter. MetaMapAdapter 
  
   MetaMapAlgorithm   - Class in  org.metaqtl.algo  This class is dedicated to the construction of a consensus
 genetic map from a set of genetic maps based on a Weigthed
 Least Square approach.   MetaMapAlgorithm(IBioGenome[], IBioGenome, IBioLocus[], MetaDico)   - 
Constructor for class org.metaqtl.algo. MetaMapAlgorithm 
  
   MetaMapSummaryFactory   - Class in  org.metaqtl.factory  A factory to write out the information on a   MetaMap  .   MetaMapSummaryFactory()   - 
Constructor for class org.metaqtl.factory. MetaMapSummaryFactory 
  
   metaQtlAnalysis   - 
Variable in class org.metaqtl. Chromosome 
 The results of a meta-analysis of QTL.
   MetaQtlAnalysis   - Class in  org.metaqtl  A QTL meta analysis consists in a clustering of QTL positions over
 chromosomes for different group of traits.   MetaQtlAnalysis(int)   - 
Constructor for class org.metaqtl. MetaQtlAnalysis 
 Creates a new instance of a  MetaQtlAnalysis 
   MetaQtlAnalysisAdapter   - Class in  org.metaqtl.adapter      MetaQtlAnalysisAdapter()   - 
Constructor for class org.metaqtl.adapter. MetaQtlAnalysisAdapter 
  
   MetaQtlAnalysisFactory   - Class in  org.metaqtl.factory  A factory to deal with the result of a QTL meta-analysis.   MetaQtlAnalysisFactory()   - 
Constructor for class org.metaqtl.factory. MetaQtlAnalysisFactory 
  
   MetaQtlAnalysisModelFactory   - Class in  org.metaqtl.factory      MetaQtlAnalysisModelFactory()   - 
Constructor for class org.metaqtl.factory. MetaQtlAnalysisModelFactory 
  
   MetaQtlAnalysisSummaryFactory   - Class in  org.metaqtl.factory  A factory to deal with QTL meta-analysis result.   MetaQtlAnalysisSummaryFactory()   - 
Constructor for class org.metaqtl.factory. MetaQtlAnalysisSummaryFactory 
  
   MetaQtlData   - Class in  org.metaqtl  QTL meta analysis first requires to gather the QTL according
 to the group trait they belong and then to format the QTL data
 into data points to perfom clustering.   MetaQtlData(Qtl[])   - 
Constructor for class org.metaqtl. MetaQtlData 
 Create a new instance of  MetaQtlData  which
 will represent the given array of  Qtl .
   MetaQtlModel   - Class in  org.metaqtl      MetaQtlModel()   - 
Constructor for class org.metaqtl. MetaQtlModel 
  
   MetaQtlModel(int)   - 
Constructor for class org.metaqtl. MetaQtlModel 
  
   MetaQtlModelFactory   - Class in  org.metaqtl.factory      MetaQtlModelFactory()   - 
Constructor for class org.metaqtl.factory. MetaQtlModelFactory 
  
   MetaQtlResult   - Class in  org.metaqtl  A  MetaQtlResult  is used to store the 
 results of a QTL meta analysis which has been performed
 for a given trait group on a given chromosome.   MetaQtlResult()   - 
Constructor for class org.metaqtl. MetaQtlResult 
  
   MetaQtlResult(String, int)   - 
Constructor for class org.metaqtl. MetaQtlResult 
  
   METHOD   - 
Static variable in class org.metaqtl.algo. HClustAlgorithm 
 A global variable to control the custering
 method.
   mfc   - 
Variable in class org.metaqtl. Chromosome 
 The mapping function for this chromosome
   mir   - 
Variable in class org.metaqtl. EMCriteria 
  
   missing_sd_mode   - 
Variable in class org.metaqtl.algo. ClustAlgorithm 
 This global variable defines the mode of imputation
 for the QTL for which the standard deviation of the position
 is not available.
   MMapView   - Class in  org.metaqtl.main      MMapView()   - 
Constructor for class org.metaqtl.main. MMapView 
  
   mode   - 
Variable in class org.metaqtl. QtlPartition 
 The mode of representation
   models   - 
Variable in class org.metaqtl. MetaQtlModel 
 The number of Qtl per chromosome
 and per trait.
   mqtls   - 
Variable in class org.metaqtl. Chromosome 
 The meta Qtls on this chromosome.
   MQTLView   - Class in  org.metaqtl.main      MQTLView()   - 
Constructor for class org.metaqtl.main. MQTLView 
  
   mr   - 
Variable in class org.metaqtl. Chromosome 
 The adjacent recombination rate between 
 ordered markers
   mrkNames   - 
Variable in class org.metaqtl. Chromosome 
 The ordered marker names
   mrkNames   - 
Variable in class org.metaqtl. MetaChrom 
 The ordered marker names.
   mrkOc   - 
Variable in class org.metaqtl. MetaChrom 
 The number of occurence per marker oved chromosomes.
   mStep(double[], double[], EMResult, double)   - 
Static method in class org.metaqtl.algo. EMAlgorithm 
 The Maximization step : here this step is straighforward and 
 simple analytical formula are applied to obtain the new parameter
 estimates.
   mu   - 
Variable in class org.metaqtl. EMResult 
 The means of the clusters.
   MultiFitResult   - Class in  org.metaqtl  This class represents a result of a multilinear regression
 analysis.   MultiFitResult(int, int)   - 
Constructor for class org.metaqtl. MultiFitResult 
 Creates a new instance of  MultiFitResult 
 with the given number of observations  N 
 and parameters  M .
   mut   - 
Variable in class org.metaqtl. Chromosome 
 The mapping unit for this chromosome
 
 
    
 N  
 
   n   - 
Variable in class org.metaqtl. EMResult 
 The number of data points.
   n   - 
Variable in class org.metaqtl. MultiFitResult 
 The number of observations without missing data.
   name   - 
Variable in class org.metaqtl.bio.entity.bean. BioEntityBean 
  
   name   - 
Variable in class org.metaqtl.bio.entity.bean. PropertyBean 
  
   name   - 
Variable in class org.metaqtl.bio.entity. BioEntity 
  
   name   - 
Variable in class org.metaqtl.bio.entity. BioEntityCollection 
  
   name   - 
Variable in class org.metaqtl. ChromCluster 
 The name of the chromosome cluster.
   name   - 
Variable in class org.metaqtl. Chromosome 
 The name of the chromosome.
   name   - 
Variable in class org.metaqtl.graph. QtlUnit 
  
   name   - 
Variable in class org.metaqtl. MetaChrom 
 The name of the meta chrom.
   name   - 
Variable in class org.metaqtl. Qtl 
 The name of the QTL.
   name   - 
Variable in class org.metaqtl. Qtl.QTLCross 
 The name of the cross design.
   name   - 
Variable in class org.metaqtl. Qtl.QTLTrait 
 The name of the trait.
   nc   - 
Variable in class org.metaqtl. MetaChrom 
 The number of chromosomes implied.
   nchr   - 
Variable in class org.metaqtl. MetaMap 
 The number of meta chromosmes.
   nchr   - 
Variable in class org.metaqtl. MetaQtlAnalysis 
 The number of chromosomes on which the meta-analysis
 has been performed.
   ncs   - 
Variable in class org.metaqtl. CMarkerSequence 
 The number of common sequences.
   needToBeRescaled(IBioGenome)   - 
Static method in class org.metaqtl.util. GeneticMapProperties 
 Returns true if the given map need to be rescaled - due
 to the mapping extansion for some pedigree designs.
   NEG_COMMON   - 
Static variable in class org.metaqtl.graph. ChromLayer 
  
   NEG_COMMON_COLOR   - 
Static variable in class org.metaqtl.graph. MetaGraphPar 
  
   newBioEntity(int)   - 
Static method in class org.metaqtl.bio.entity. BioEntity 
  
   newCrossPopulation(int, String, IBioEntity)   - 
Static method in class org.metaqtl.bio.entity. Population 
  
   newEstimatedLocusPosition(double, double)   - 
Static method in class org.metaqtl.bio.util. LGroupPosition 
  
   newLocus(int)   - 
Static method in class org.metaqtl.bio.entity. Locus 
 Returns a new Locus
 from the given locus type.
   newLocus(String)   - 
Static method in class org.metaqtl.bio.entity. Locus 
 Returns a new Locus
 from the given locus type.
   newStaticLocusPosition(double)   - 
Static method in class org.metaqtl.bio.util. LGroupPosition 
  
   nextValues(Hashtable)   - 
Method in class org.metaqtl.bio.util. TabulatedWriter 
 Prints one line of tabulated values given the hashtable which links
 values and keys.
   nextValues(String[])   - 
Method in class org.metaqtl.bio.util. TabulatedWriter 
 Print one line of tabulated values by assuming that they are in the same
 order than the keys.
   ni   - 
Variable in class org.metaqtl. ChromCluster 
 The total number of marker intervals.
   ni   - 
Variable in class org.metaqtl. MetaChrom 
 The number of marker intervals implied in this consensus.
   nidx   - 
Variable in class org.metaqtl. TreeNode 
 The indices of the sub-nodes
   nm   - 
Variable in class org.metaqtl. Chromosome 
 The number of markers.
   nm   - 
Variable in class org.metaqtl. MetaChrom 
 The number of markers.
   nmc   - 
Variable in class org.metaqtl. ChromCluster 
 Total number of common marker between clusters
   nmc   - 
Variable in class org.metaqtl. CMarkerSequence 
 The number of common marker between chromosomes
   nmc   - 
Variable in class org.metaqtl. MetaChrom 
 The number of marker per implied chromosome
   nmqc   - 
Variable in class org.metaqtl. MetaQtlResult 
 The number of meta qtl clusterings.
   nodes   - 
Variable in class org.metaqtl. Tree 
 The nodes of the tree.
   np   - 
Variable in class org.metaqtl. QtlPartition 
 The number of partition.
   nqtl   - 
Variable in class org.metaqtl. MetaQtlResult 
 The number of QTL
   nqtl   - 
Variable in class org.metaqtl. QtlPartition 
 The number of Qtl.
   ntg   - 
Variable in class org.metaqtl. MetaQtlData 
 The number of trait group.
   ntgQtls   - 
Variable in class org.metaqtl. MetaQtlData 
 The number of QTL in each
 trait group.
   NumberFormat   - Class in  org.metaqtl.bio.util      NumberFormat()   - 
Constructor for class org.metaqtl.bio.util. NumberFormat 
  
   NumericalUtilities   - Class in  org.metaqtl.numrec  Class Description Here   NumericalUtilities()   - 
Constructor for class org.metaqtl.numrec. NumericalUtilities 
  
   NumericalUtilities.Function   - Interface in  org.metaqtl.numrec      NumericalUtilities.NRFunction   - Interface in  org.metaqtl.numrec    
 
    
 O  
 
   o_mrkIdxL   - 
Variable in class org.metaqtl. Qtl.QTLProj 
 The original indice of the left
 flanking marker.
   o_mrkIdxR   - 
Variable in class org.metaqtl. Qtl.QTLProj 
 The original indice of the right 
 flanking marker.
   OCCURENCE   - 
Static variable in interface org.metaqtl. IBioLocusProperties 
 The key for the occurence of markers in a consensus map
   ocov   - 
Variable in class org.metaqtl. EMResult 
 The observed information (just for means).
   OffspringManager   - Class in  org.metaqtl.util  Class Description Here   OffspringManager()   - 
Constructor for class org.metaqtl.util. OffspringManager 
  
   OffspringManager.HaldaneWaddingtonProportion   - Class in  org.metaqtl.util      OffspringManager.HaldaneWaddingtonProportion()   - 
Constructor for class org.metaqtl.util. OffspringManager.HaldaneWaddingtonProportion 
  
   olog   - 
Variable in class org.metaqtl. EMResult 
 The observed loglikelihood.
   ontology   - 
Variable in class org.metaqtl.algo. ClustAlgorithm 
 The optional trait ontology to use to group Qtl.
   Ontology   - Class in  org.metaqtl.bio.entity      Ontology()   - 
Constructor for class org.metaqtl.bio.entity. Ontology 
  
   ONTOLOGY   - 
Static variable in interface org.metaqtl.bio. IBioEntityTypes 
  
   ONTOLOGY_TERM   - 
Static variable in interface org.metaqtl.bio. IBioEntityTypes 
  
   OntologyBean   - Class in  org.metaqtl.bio.entity.bean      OntologyBean()   - 
Constructor for class org.metaqtl.bio.entity.bean. OntologyBean 
  
   OntologyBeanAdapter   - Class in  org.metaqtl.bio.entity.adapter      OntologyBeanAdapter()   - 
Constructor for class org.metaqtl.bio.entity.adapter. OntologyBeanAdapter 
  
   OntologyTabFactory   - Class in  org.metaqtl.bio.entity.factory  A factory to deal with   Ontology  .   OntologyTabFactory()   - 
Constructor for class org.metaqtl.bio.entity.factory. OntologyTabFactory 
  
   OntologyTerm   - Class in  org.metaqtl.bio.entity      OntologyTerm()   - 
Constructor for class org.metaqtl.bio.entity. OntologyTerm 
  
   OntologyTermBean   - Class in  org.metaqtl.bio.entity.bean      OntologyTermBean()   - 
Constructor for class org.metaqtl.bio.entity.bean. OntologyTermBean 
  
   OntologyTermBeanAdapter   - Class in  org.metaqtl.bio.entity.adapter      OntologyTermBeanAdapter()   - 
Constructor for class org.metaqtl.bio.entity.adapter. OntologyTermBeanAdapter 
  
   org.metaqtl   - package org.metaqtl     org.metaqtl.adapter   - package org.metaqtl.adapter     org.metaqtl.algo   - package org.metaqtl.algo     org.metaqtl.bio   - package org.metaqtl.bio     org.metaqtl.bio.entity   - package org.metaqtl.bio.entity     org.metaqtl.bio.entity.adapter   - package org.metaqtl.bio.entity.adapter     org.metaqtl.bio.entity.bean   - package org.metaqtl.bio.entity.bean     org.metaqtl.bio.entity.factory   - package org.metaqtl.bio.entity.factory     org.metaqtl.bio.util   - package org.metaqtl.bio.util     org.metaqtl.factory   - package org.metaqtl.factory     org.metaqtl.graph   - package org.metaqtl.graph     org.metaqtl.main   - package org.metaqtl.main     org.metaqtl.numrec   - package org.metaqtl.numrec     org.metaqtl.util   - package org.metaqtl.util   
 
    
 P  
 
   p1   - 
Variable in class org.metaqtl.graph. ChromLayer.CMrkTickPoint 
  
   p2   - 
Variable in class org.metaqtl.graph. ChromLayer.CMrkTickPoint 
  
   PaletteColor   - Class in  org.metaqtl.graph      PaletteColor()   - 
Constructor for class org.metaqtl.graph. PaletteColor 
  
   parent   - 
Variable in class org.metaqtl.bio.entity. BioEntity 
  
   parent   - 
Variable in class org.metaqtl.bio.entity. BioEntityCollection 
  
   parent   - 
Variable in class org.metaqtl. TreeNode 
 The parent of the node
   parents   - 
Variable in class org.metaqtl.bio.entity. Population 
  
   parse(String[])   - 
Method in class org.metaqtl.main. CmdLineParser 
 Extract the options and non-option arguments from the given
 list of command-line arguments.
   parse(String[], Locale)   - 
Method in class org.metaqtl.main. CmdLineParser 
 Extract the options and non-option arguments from the given
 list of command-line arguments.
   parseChromList(String)   - 
Static method in class org.metaqtl.main. MetaMain 
  
   parseCmdLine(String[])   - 
Method in class org.metaqtl.main. MetaMain 
  
   parseCross(String)   - 
Static method in class org.metaqtl.bio.util. BioCrossTypes 
 Parse the given string and return if valid the type of
 cross and the number of generations.
   parseDouble(String)   - 
Static method in class org.metaqtl.bio.util. NumberFormat 
 This method parse the given string to a double value.
   parser   - 
Variable in class org.metaqtl.main. MetaMain 
  
   parseType(String)   - 
Static method in class org.metaqtl.bio.util. BioLocusTypes 
  
   parseValue(String, Locale)   - 
Method in class org.metaqtl.main. CmdLineParser.Option.DoubleOption 
  
   parseValue(String, Locale)   - 
Method in class org.metaqtl.main. CmdLineParser.Option.IntegerOption 
  
   parseValue(String, Locale)   - 
Method in class org.metaqtl.main. CmdLineParser.Option.LongOption 
  
   parseValue(String, Locale)   - 
Method in class org.metaqtl.main. CmdLineParser.Option 
 Override to extract and convert an option value passed on the
 command-line
   parseValue(String, Locale)   - 
Method in class org.metaqtl.main. CmdLineParser.Option.StringOption 
  
   partition   - 
Variable in class org.metaqtl.graph. QtlUnit 
  
   path   - 
Variable in class org.metaqtl.graph. ChromLayer.CMrkTickPath 
  
   pi   - 
Variable in class org.metaqtl. EMResult 
 The mixing proportions.
   pnames   - 
Variable in class org.metaqtl. QtlPartition 
 The partition names.
   population   - 
Variable in class org.metaqtl.bio.entity. Individual 
  
   Population   - Class in  org.metaqtl.bio.entity  Class Description Here   Population()   - 
Constructor for class org.metaqtl.bio.entity. Population 
  
   Population(String, IBioEntity)   - 
Constructor for class org.metaqtl.bio.entity. Population 
  
   POPULATION   - 
Static variable in interface org.metaqtl.bio. IBioEntityTypes 
 The integer code for Population entity
   pos   - 
Variable in class org.metaqtl.graph. QtlUnit 
  
   POS_COMMON   - 
Static variable in class org.metaqtl.graph. ChromLayer 
  
   POS_COMMON_COLOR   - 
Static variable in class org.metaqtl.graph. MetaGraphPar 
  
   position   - 
Variable in class org.metaqtl.bio.entity.bean. LocusBean 
  
   position   - 
Variable in class org.metaqtl.bio.entity. Locus 
  
   position   - 
Variable in class org.metaqtl. Qtl 
 The QTL most probable location on the chromosome.
   printHelp()   - 
Method in class org.metaqtl.main. A2Xml 
  
   printHelp()   - 
Method in class org.metaqtl.main. ConsMap 
  
   printHelp()   - 
Method in class org.metaqtl.main. InfoMap 
  
   printHelp()   - 
Method in class org.metaqtl.main. MapView 
  
   printHelp()   - 
Method in class org.metaqtl.main. MetaDB 
  
   printHelp()   - 
Method in class org.metaqtl.main. MetaMain 
  
   printHelp()   - 
Method in class org.metaqtl.main. MMapView 
  
   printHelp()   - 
Method in class org.metaqtl.main. MQTLView 
  
   printHelp()   - 
Method in class org.metaqtl.main. QTLClust 
  
   printHelp()   - 
Method in class org.metaqtl.main. QTLClustInfo 
  
   printHelp()   - 
Method in class org.metaqtl.main. QTLModel 
  
   printHelp()   - 
Method in class org.metaqtl.main. QTLProj 
  
   printHelp()   - 
Method in class org.metaqtl.main. QTLTree 
  
   printHelp()   - 
Method in class org.metaqtl.main. Xml2A 
  
   printImgFormat(int)   - 
Static method in class org.metaqtl.main. MetaMain 
  
   printLicense(String, String)   - 
Static method in class org.metaqtl.main. MetaMain 
  
   printUsage()   - 
Method in class org.metaqtl.main. A2Xml 
  
   printUsage()   - 
Method in class org.metaqtl.main. ConsMap 
  
   printUsage()   - 
Method in class org.metaqtl.main. InfoMap 
  
   printUsage()   - 
Method in class org.metaqtl.main. MapView 
  
   printUsage()   - 
Method in class org.metaqtl.main. MetaDB 
  
   printUsage()   - 
Method in class org.metaqtl.main. MetaMain 
  
   printUsage()   - 
Method in class org.metaqtl.main. MMapView 
  
   printUsage()   - 
Method in class org.metaqtl.main. MQTLView 
  
   printUsage()   - 
Method in class org.metaqtl.main. QTLClust 
  
   printUsage()   - 
Method in class org.metaqtl.main. QTLClustInfo 
  
   printUsage()   - 
Method in class org.metaqtl.main. QTLModel 
  
   printUsage()   - 
Method in class org.metaqtl.main. QTLProj 
  
   printUsage()   - 
Method in class org.metaqtl.main. QTLTree 
  
   printUsage()   - 
Method in class org.metaqtl.main. Xml2A 
  
   proba   - 
Variable in class org.metaqtl. Chromosome 
 Probabilities defined along the chromosome.
   proba   - 
Variable in class org.metaqtl. QtlPartition 
 The probabilities of each Qtl to
 belong to each partition.
   PROBA_BIN   - 
Static variable in class org.metaqtl.graph. MetaGraphPar 
  
   PROBA_FROM_COLOR   - 
Static variable in class org.metaqtl.graph. MetaGraphPar 
  
   PROBA_PALETTE   - 
Static variable in class org.metaqtl.graph. MetaGraphPar 
  
   PROBA_TO_COLOR   - 
Static variable in class org.metaqtl.graph. MetaGraphPar 
  
   proj   - 
Variable in class org.metaqtl. Qtl 
 The QTL projection properties.
   properties   - 
Variable in class org.metaqtl.bio.entity.bean. BioEntityBean 
  
   properties   - 
Variable in class org.metaqtl.bio.entity. BioEntity 
  
   properties   - 
Variable in class org.metaqtl.bio.entity. BioEntityCollection 
  
   PropertyBean   - Class in  org.metaqtl.bio.entity.bean  Class Description Here   PropertyBean()   - 
Constructor for class org.metaqtl.bio.entity.bean. PropertyBean 
  
   PropertyBean(String, String)   - 
Constructor for class org.metaqtl.bio.entity.bean. PropertyBean 
  
   PVALUE   - 
Static variable in interface org.metaqtl. IBioLGroupProperties 
 The key for the p-value.
   pvalue   - 
Variable in class org.metaqtl. MetaChrom 
 The p-value of this chromosome.
   pvalue   - 
Variable in class org.metaqtl. MultiFitResult 
 The p-value of the model.
   PYTHAG(double, double)   - 
Static method in class org.metaqtl.numrec. NumericalUtilities 
 Computes sqrt(a^2+b^2)
 
 
    
 Q  
 
   QTL   - Class in  org.metaqtl.bio.entity  Class Description Here   QTL()   - 
Constructor for class org.metaqtl.bio.entity. QTL 
  
   QTL(String, IBioEntity)   - 
Constructor for class org.metaqtl.bio.entity. QTL 
  
   QTL   - 
Static variable in interface org.metaqtl.bio. IBioLocus 
  
   Qtl   - Class in  org.metaqtl  A QTL (Quantitative Trait Loci)   Qtl()   - 
Constructor for class org.metaqtl. Qtl 
  
   Qtl.QTLCross   - Class in  org.metaqtl  This class stores the informations on the cross
 design used to detect the QTL.   Qtl.QTLCross()   - 
Constructor for class org.metaqtl. Qtl.QTLCross 
  
   Qtl.QTLProj   - Class in  org.metaqtl  This class stores the QTL projection
 properties.   Qtl.QTLProj()   - 
Constructor for class org.metaqtl. Qtl.QTLProj 
  
   Qtl.QTLStats   - Class in  org.metaqtl  This class stores the usual statistics for the QTL.   Qtl.QTLStats()   - 
Constructor for class org.metaqtl. Qtl.QTLStats 
  
   Qtl.QTLSupport   - Class in  org.metaqtl  This class stores information
 on the QTL Confidence Interval   Qtl.QTLSupport()   - 
Constructor for class org.metaqtl. Qtl.QTLSupport 
  
   Qtl.QTLTrait   - Class in  org.metaqtl  This class stores the trait description of a QTL   Qtl.QTLTrait()   - 
Constructor for class org.metaqtl. Qtl.QTLTrait 
  
   QTL_CI_FROM   - 
Static variable in interface org.metaqtl. IBioLocusProperties 
 The key for the QTL confidence interval lower boundary.
   QTL_CI_LOD_DECREASE   - 
Static variable in interface org.metaqtl. IBioLocusProperties 
 The key for the QTL confidence interval LOD decrease.
   QTL_CI_TO   - 
Static variable in interface org.metaqtl. IBioLocusProperties 
 THe key for the QTL confidence interval upper boundary.
   QTL_CI_WIDTH   - 
Static variable in class org.metaqtl.graph. MetaGraphPar 
  
   QTL_CI_WIDTH_CEX   - 
Static variable in class org.metaqtl.graph. MetaGraphPar 
  
   QTL_CROSS_NAME   - 
Static variable in interface org.metaqtl. IBioLocusProperties 
 The key for the QTL cross name.
   QTL_CROSS_SIZE   - 
Static variable in interface org.metaqtl. IBioLocusProperties 
 The key for the QTL cross size.
   QTL_CROSS_TYPE   - 
Static variable in interface org.metaqtl. IBioLocusProperties 
 The key for the QTL cross type.
   QTL_HSPACE   - 
Static variable in class org.metaqtl.graph. MetaGraphPar 
 The horizontal space between Qtl shapes.
   QTL_NAME_COLOR   - 
Static variable in class org.metaqtl.graph. MetaGraphPar 
 The color of the QTL names.
   QTL_NAME_FONT   - 
Static variable in class org.metaqtl.graph. MetaGraphPar 
 The font used for the Qtl names.
   QTL_NAME_SPACE   - 
Static variable in class org.metaqtl.graph. MetaGraphPar 
  
   QTL_NAME_VSPACE_CEX   - 
Static variable in class org.metaqtl.graph. MetaGraphPar 
 The space magnification between the Qtl name and the Qtl confidence
 interval.
   QTL_PALETTE   - 
Static variable in class org.metaqtl.graph. MetaGraphPar 
 The color palete for the QTL partition.
   QTL_PCH_SIZE   - 
Static variable in class org.metaqtl.graph. MetaGraphPar 
  
   QTL_POS_HEIGHT_CEX   - 
Static variable in class org.metaqtl.graph. MetaGraphPar 
  
   QTL_POS_WIDTH_CEX   - 
Static variable in class org.metaqtl.graph. MetaGraphPar 
  
   QTL_PROB_WIDTH   - 
Static variable in class org.metaqtl.graph. MetaGraphPar 
  
   QTL_PROJ_INTSCALE   - 
Static variable in interface org.metaqtl. IBioLocusProperties 
 The key for the QTL flanking marker interval projection
 rate.
   QTL_PROJ_MAPSCALE   - 
Static variable in interface org.metaqtl. IBioLocusProperties 
 The key for the QTL confidence interval projection rate.
   QTL_PROJ_ORIGIN   - 
Static variable in interface org.metaqtl. IBioLocusProperties 
 The key for the map of origin of the QTL.
   QTL_PROJ_SHAREFLANKING   - 
Static variable in interface org.metaqtl. IBioLocusProperties 
 The key for the QTL flanking marker interval order preservation.
   QTL_PROJ_SWAPFLANKING   - 
Static variable in interface org.metaqtl. IBioLocusProperties 
 The key for the QTL flanking marker interval reverse order.
   QTL_STATS_ADDITIVE   - 
Static variable in interface org.metaqtl. IBioLocusProperties 
 The key for the QTL additive effect.
   QTL_STATS_DOMINANCE   - 
Static variable in interface org.metaqtl. IBioLocusProperties 
 The key for the QTL dominance effect.
   QTL_STATS_RSQUARE   - 
Static variable in interface org.metaqtl. IBioLocusProperties 
 The key for the QTL rsquare.
   QTL_TICK_WIDTH_CEX   - 
Static variable in class org.metaqtl.graph. MetaGraphPar 
 The magnification for the tick of the QTL.
   QTL_TRAIT_GROUP   - 
Static variable in interface org.metaqtl. IBioLocusProperties 
 The key for the QTL trait group.
   QTL_TRAIT_NAME   - 
Static variable in interface org.metaqtl. IBioLocusProperties 
 The key for the QTL trait name.
   QTL_TRAIT_UNIT   - 
Static variable in interface org.metaqtl. IBioLocusProperties 
 The key for the QTL trait unit.
   QTL_TREE_SCALE   - 
Static variable in class org.metaqtl.graph. MetaGraphPar 
  
   QTL_TREE_SPACE   - 
Static variable in class org.metaqtl.graph. MetaGraphPar 
  
   QTL_VSPACE   - 
Static variable in class org.metaqtl.graph. MetaGraphPar 
 The vertical space between Qtl shapes.
   QtlAdapter   - Class in  org.metaqtl.adapter  This class defines some usefull methods to adapt   Qtl  
 object into other kinds of objects or the inverse.   QtlAdapter()   - 
Constructor for class org.metaqtl.adapter. QtlAdapter 
  
   qtlByChrom   - 
Variable in class org.metaqtl. MetaQtlAnalysis 
 The matrix of qtl by chromosome
   qtlCI   - 
Variable in class org.metaqtl. QtlPartition 
 The Qtl confidence intervals.
   QTLClust   - Class in  org.metaqtl.main      QTLClust()   - 
Constructor for class org.metaqtl.main. QTLClust 
  
   QtlClustAlgorithm   - Class in  org.metaqtl.algo  This class defines methods to perform a QTL meta-analysis
 on a genetic map by applying a clustering of QTL
 based on a gaussian mixture model and the EM-algorihtm.   QtlClustAlgorithm(IBioGenome, IBioOntology)   - 
Constructor for class org.metaqtl.algo. QtlClustAlgorithm 
  
   QtlClustAlgorithm(IBioGenome, String, IBioOntology)   - 
Constructor for class org.metaqtl.algo. QtlClustAlgorithm 
  
   QTLClustInfo   - Class in  org.metaqtl.main      QTLClustInfo()   - 
Constructor for class org.metaqtl.main. QTLClustInfo 
  
   qtlIdx   - 
Variable in class org.metaqtl. MetaQtlResult 
 The indices of QTL which are implied
 in the analysis.
   QtlLayer   - Class in  org.metaqtl.graph      QtlLayer(double, double)   - 
Constructor for class org.metaqtl.graph. QtlLayer 
  
   QtlLayerBuffer   - Class in  org.metaqtl.graph      QtlLayerBuffer()   - 
Constructor for class org.metaqtl.graph. QtlLayerBuffer 
  
   QTLLOCUS   - 
Static variable in interface org.metaqtl.bio. IBioEntityTypes 
  
   QTLModel   - Class in  org.metaqtl.main      QTLModel()   - 
Constructor for class org.metaqtl.main. QTLModel 
  
   qtlNames   - 
Variable in class org.metaqtl. QtlPartition 
 The qtl names
   qtlPart   - 
Variable in class org.metaqtl.graph. ChromGraph 
 The Qtl partition
   QtlPartition   - Class in  org.metaqtl      QtlPartition()   - 
Constructor for class org.metaqtl. QtlPartition 
  
   QtlPartitionAdapter   - Class in  org.metaqtl.adapter  This class defines some methods to adapt complient
 objects into a   QtlPartition   object.   QtlPartitionAdapter()   - 
Constructor for class org.metaqtl.adapter. QtlPartitionAdapter 
  
   QtlPartitionLayer   - Class in  org.metaqtl.graph      QtlPartitionLayer(double, double)   - 
Constructor for class org.metaqtl.graph. QtlPartitionLayer 
  
   qtlPos   - 
Variable in class org.metaqtl. QtlPartition 
 The Qtl positions
   QTLProj   - Class in  org.metaqtl.main      QTLProj()   - 
Constructor for class org.metaqtl.main. QTLProj 
  
   QtlProjAlgorithm   - Class in  org.metaqtl.algo  This class defines some methods to project QTL positions from 
 a genetic map to another.   QtlProjAlgorithm(IBioGenome[], IBioGenome, IBioLocus[])   - 
Constructor for class org.metaqtl.algo. QtlProjAlgorithm 
 Creates a new instance of  QtlProjAlgorithm  for
 the given configuration of projection.
   qtls   - 
Variable in class org.metaqtl. Chromosome 
 The QTLs located on this chromosome.
   qtls   - 
Variable in class org.metaqtl. MetaQtlData 
 The QTL.
   QtlShape   - Class in  org.metaqtl.graph      QtlShape()   - 
Constructor for class org.metaqtl.graph. QtlShape 
  
   qtlShapes   - 
Variable in class org.metaqtl.graph. QtlLayer 
 The Qtl shapes.
   QTLTree   - Class in  org.metaqtl.main      QTLTree()   - 
Constructor for class org.metaqtl.main. QTLTree 
  
   QtlTreeAlgorithm   - Class in  org.metaqtl.algo  This class defines methods to perform agglomerative
 hierarchical QTL clustering.   QtlTreeAlgorithm(IBioGenome, IBioOntology)   - 
Constructor for class org.metaqtl.algo. QtlTreeAlgorithm 
  
   QtlTreeLayer   - Class in  org.metaqtl.graph      QtlTreeLayer(double, double)   - 
Constructor for class org.metaqtl.graph. QtlTreeLayer 
  
   QtlUnit   - Class in  org.metaqtl.graph      QtlUnit()   - 
Constructor for class org.metaqtl.graph. QtlUnit 
  
 
 
    
 R  
 
   r2R()   - 
Method in class org.metaqtl. Chromosome 
  
   r2R(int, int)   - 
Method in class org.metaqtl. Chromosome 
 Re-scale the chromosome assuming that the distances
 and recombination fractions are based on the frequency of
 recombinant genotypes R instead of the real recombination
 fraction r.
   R2r()   - 
Method in class org.metaqtl. Chromosome 
  
   R2r(int, int)   - 
Method in class org.metaqtl. Chromosome 
 Re-scale the chromosome assuming that the distances
 and recombination fractions are based on the frequency of
 recombinant genotypes R instead of the real recombination
 fraction r.
   R2r(double, int, int)   - 
Static method in class org.metaqtl.util. OffspringManager 
 This routine returns the recombination rate r for a given 
 value of the propertion of recombibant genotypes R according
 to the cross design.
   r2R(double, int, int)   - 
Static method in class org.metaqtl.util. OffspringManager 
 This routine returns the recombination rate r for a given 
 value of the propertion of recombibant genotypes R according
 to the cross design.
   rate   - 
Variable in class org.metaqtl. EMResult 
 The quadratric rate of convergence
 of the algorithm.
   read(Reader)   - 
Static method in class org.metaqtl.factory. DubiousLocusFactory 
 This routine reads the list of dubious markers from
 the given file location and returns if succeed an
 array of  IBioLocus  objects.
   read(BufferedReader)   - 
Static method in class org.metaqtl.factory. EMResultFactory 
  
   read(Reader)   - 
Static method in class org.metaqtl.factory. MapDBFactory 
 This method reads a file formated as follows :
 
 map    property1  property2  property3 ...
   read(Reader)   - 
Static method in class org.metaqtl.factory. MetaDicoFactory 
  
   read(InputStream)   - 
Static method in class org.metaqtl.factory. MetaQtlAnalysisFactory 
  
   read(Reader)   - 
Static method in class org.metaqtl.factory. MetaQtlAnalysisFactory 
  
   read(Reader)   - 
Static method in class org.metaqtl.factory. MetaQtlModelFactory 
 This method reads a file formated as follows :
 
 Chromosome \t Trait \t Model
   read(Reader)   - 
Static method in class org.metaqtl.factory. TreeFactory 
  
   read(String)   - 
Static method in class org.metaqtl.factory. TreeFactory 
  
   read_newick(Reader)   - 
Static method in class org.metaqtl.factory. TreeFactory 
  
   read_newick(String)   - 
Static method in class org.metaqtl.factory. TreeFactory 
  
   rebuildPartition(String[])   - 
Method in class org.metaqtl. QtlPartition 
  
   recombination(double, int, int)   - 
Static method in class org.metaqtl.util. MappingFunction 
 Returns the recombination rate from the distance.
   removeAllele(String)   - 
Method in class org.metaqtl.bio.entity. AlleleContainer 
  
   removeAllele(String)   - 
Method in interface org.metaqtl.bio. IBioLocus 
  
   removeEntity(String)   - 
Method in class org.metaqtl.bio.entity. BioEntityContainer 
  
   removeGroup(String)   - 
Method in class org.metaqtl.bio.entity. GroupContainer 
  
   removeGroup(String)   - 
Method in interface org.metaqtl.bio. IBioGenome 
  
   removeIndividual(String)   - 
Method in class org.metaqtl.bio.entity. IndividualContainer 
  
   removeIndividual(String)   - 
Method in interface org.metaqtl.bio. IBioPopulation 
  
   removeLocus(String)   - 
Method in class org.metaqtl.bio.entity. LocusContainer 
  
   removeLocus(String)   - 
Method in interface org.metaqtl.bio. IBioLGroup 
  
   removeLocus(IBioGenome[], String)   - 
Static method in class org.metaqtl.main. MetaMain 
  
   result   - 
Variable in class org.metaqtl.algo. ClustAlgorithm 
 The result of the clustering.
   resultByChrom   - 
Variable in class org.metaqtl. MetaQtlAnalysis 
 The matrix of results where the rows are the chromosomes.
   RF   - 
Static variable in interface org.metaqtl.bio. IBioCrossTypes 
  
   RIGHT_LABEL   - 
Static variable in class org.metaqtl.graph. ChromLayer 
  
   RIGHT_LABEL   - 
Static variable in class org.metaqtl.graph. ChromShape 
  
   RILSe   - 
Static variable in interface org.metaqtl.bio. IBioCrossTypes 
  
   RILSi   - 
Static variable in interface org.metaqtl.bio. IBioCrossTypes 
  
   root   - 
Variable in class org.metaqtl.bio.entity.bean. OntologyBean 
 The root of the ontology.
   root   - 
Variable in class org.metaqtl. Tree 
 The root of the tree.
   rsd   - 
Variable in class org.metaqtl. MetaChrom 
 The standardized residual between original marker intervals
 and those of the meta chromosome.
   rsd   - 
Variable in class org.metaqtl. MultiFitResult 
 The residual standard deviations.
   rsquare   - 
Variable in class org.metaqtl. Qtl.QTLStats 
 The r-square, i.e the proportion of variance 
 explained by the QTL.
   rtsafe(NumericalUtilities.NRFunction, double, double, double)   - 
Static method in class org.metaqtl.numrec. NumericalUtilities 
  
   run(Chromosome, Chromosome)   - 
Static method in class org.metaqtl.algo. CMSAlgorithm 
 This methods find the common marker sequences between the 
 two given chromosomes.
   run(double[], double[])   - 
Static method in class org.metaqtl.algo. HClustAlgorithm 
 This method performs a agglomerative clustering
 of the given data points where  x  is the 
 vector of observed values and  sd  the
 vector of their standard deviations.
   run()   - 
Method in class org.metaqtl.algo. MetaMapAlgorithm 
 This method builds a consensus genetic map from the given
 array of  maps .
   run()   - 
Method in class org.metaqtl.algo. QtlClustAlgorithm 
 This method performs a QTL meta-analysis on each chromosome 
 of the given map.
   run()   - 
Method in class org.metaqtl.algo. QtlProjAlgorithm 
 This routine performs the projection of the QTL from the maps onto the 
 skeleton and returns the chromosomes on which the projection has been
 done.
   run()   - 
Method in class org.metaqtl.algo. QtlTreeAlgorithm 
 This method performs an agglomerative hierarchical QTL clustering 
 on each chromosome and returns the result as a   MetaQtlAnalysis  
 object.
   run()   - 
Method in interface org.metaqtl. IMetaAlgorithm 
 Run the algorithm.
 
 
    
 S  
 
   s_mrkIdxL   - 
Variable in class org.metaqtl. Qtl.QTLProj 
 The skeleton indice of the left
 flanking marker.
   s_mrkIdxR   - 
Variable in class org.metaqtl. Qtl.QTLProj 
 The skeleton indice of the right 
 flanking marker.
   scale(double)   - 
Method in class org.metaqtl.graph. ChromAxe 
 Scale the axe in y.
   scaleHeight(double)   - 
Method in class org.metaqtl.graph. ChromAxe 
  
   scaleY(double)   - 
Method in class org.metaqtl.graph. ChromAxe 
  
   sd   - 
Variable in class org.metaqtl. MetaQtlResult 
 The QTL position standard deviations.
   sd2ci(double)   - 
Static method in class org.metaqtl.numrec. NumericalUtilities 
  
   sd_mode   - 
Variable in class org.metaqtl.algo. ClustAlgorithm 
 This global variable defines the mode of compuation of
 the standard deviation of the QTL positions.
   sd_mode   - 
Variable in class org.metaqtl.algo. QtlProjAlgorithm 
 The mode of compuation of
 the standard deviation of the QTL positions.
   sd_position   - 
Variable in class org.metaqtl. Qtl 
 The standard deviation of the QTL position.
   sdd   - 
Variable in class org.metaqtl. MetaChrom 
 The standard deviation of the position in Morgan.
   selfingIndividualInformation(OffspringManager.HaldaneWaddingtonProportion)   - 
Static method in class org.metaqtl.util. OffspringManager 
 This routine returns the individual means amount of information
 for the given generation propertions for a self-fertilization
 design.
   selfingRecurrence(OffspringManager.HaldaneWaddingtonProportion, double)   - 
Static method in class org.metaqtl.util. OffspringManager 
 Given the current proportion C,D,E,F and G and the recombination
 rate r this routine computes the next generation proportions following 
 the recurrence formula of Haldane and Waddington,
 1931, Inbreeding and linkage, Genetics 16:357-374
 (see section self-fertilization p358).
   SEPARATOR   - 
Static variable in class org.metaqtl.factory. MetaQtlModelFactory 
  
   set(String, String)   - 
Static method in class org.metaqtl.graph. MetaGraphPar 
  
   setCentroid(double)   - 
Method in class org.metaqtl. TreeNode 
  
   setChildren(OntologyTerm[])   - 
Method in class org.metaqtl.bio.entity. OntologyTerm 
 Sets the children of the term.
   setChromAxe(ChromAxe)   - 
Method in class org.metaqtl.graph. QtlPartitionLayer 
  
   setChromAxe(ChromAxe)   - 
Method in class org.metaqtl.graph. QtlTreeLayer 
  
   setChromName(int, String)   - 
Method in class org.metaqtl. MetaQtlModel 
  
   setCIMiss(int)   - 
Method in class org.metaqtl.algo. ClustAlgorithm 
  
   setCIMode(int)   - 
Method in class org.metaqtl.algo. ClustAlgorithm 
  
   setClusterings(List)   - 
Method in class org.metaqtl. MetaQtlResult 
  
   setClusterings(EMResult[])   - 
Method in class org.metaqtl. MetaQtlResult 
  
   setCriterion(String)   - 
Method in class org.metaqtl. MetaQtlModel 
 Set the criterion name which was used to obtain this model
   setCrossDesign(IBioGenome, CrossPopulation)   - 
Static method in class org.metaqtl.util. GeneticMapProperties 
  
   setCrossType(int)   - 
Method in class org.metaqtl.bio.entity. CrossPopulation 
  
   setDF(double)   - 
Method in interface org.metaqtl.numrec. NumericalUtilities.Function 
 Sets the derivative of the function at x.
   setDubiousMarker(IBioLocus[])   - 
Method in class org.metaqtl. ChromCluster 
 This method sets the list of dubious marker.
   setDubiousMarker(IBioLocus[])   - 
Method in class org.metaqtl. MapCluster 
 This method sets the list of dubious marker.
   setEMEps(double)   - 
Method in class org.metaqtl.algo. QtlClustAlgorithm 
  
   setEMRanStartNumber(int)   - 
Method in class org.metaqtl.algo. QtlClustAlgorithm 
  
   setFunction(String)   - 
Method in class org.metaqtl.bio.entity. Ontology 
 Set the function of the ontology.
   setGeneration(int)   - 
Method in class org.metaqtl.bio.entity. Population 
  
   setGenome(IBioGenome)   - 
Method in class org.metaqtl.bio.entity. LocusContainer 
  
   setGenome(IBioGenome)   - 
Method in interface org.metaqtl.bio. IBioLGroup 
  
   setGroup(IBioLGroup)   - 
Method in class org.metaqtl.bio.entity. AlleleContainer 
  
   setGroup(IBioLGroup)   - 
Method in class org.metaqtl.bio.entity. Locus 
  
   setGroup(IBioLGroup)   - 
Method in interface org.metaqtl.bio. IBioLocus 
  
   setGroups(ArrayList)   - 
Method in class org.metaqtl.bio.entity.bean. GeneticMapBean 
  
   setID(long)   - 
Method in class org.metaqtl.bio.entity. OntologyTerm 
 Sets the indentifient of the term.
   setIgnore(boolean)   - 
Method in class org.metaqtl. Qtl 
 Set the status of the QTL.
   setKeys(String[])   - 
Method in class org.metaqtl.bio.util. TabulatedWriter 
  
   setLabelSide(int)   - 
Method in class org.metaqtl.graph. ChromLayer 
  
   setLabelSide(int)   - 
Method in class org.metaqtl.graph. ChromShape 
  
   setLoci(IBioLocus[])   - 
Method in class org.metaqtl.bio.entity. LocusContainer 
  
   setLoci(IBioLocus[])   - 
Method in interface org.metaqtl.bio. IBioLGroup 
  
   setLocus(ArrayList)   - 
Method in class org.metaqtl.bio.entity.bean. LGroupBean 
  
   setLocus(IBioLocus)   - 
Method in interface org.metaqtl.bio. IBioAllele 
  
   setLogger(Writer)   - 
Method in class org.metaqtl.algo. MetaAlgorithm 
  
   setLogger(OutputStream)   - 
Method in class org.metaqtl.algo. MetaAlgorithm 
  
   setLogger(Writer)   - 
Method in interface org.metaqtl. IMetaAlgorithm 
 Give a  Writer  to the algorithm
 in order to write out the log info.
   setLogger(OutputStream)   - 
Method in interface org.metaqtl. IMetaAlgorithm 
 Give a  OutputStream  to the algorithm
 in order to write out the log info.
   setMappingContext(IBioGenome, int, int)   - 
Static method in class org.metaqtl.util. GeneticMapProperties 
  
   setMarkerDico(MetaDico)   - 
Method in class org.metaqtl.algo. MetaMapAlgorithm 
  
   setMarkerDico(MetaDico)   - 
Method in class org.metaqtl. ChromCluster 
  
   setMarkerDico(MetaDico)   - 
Method in class org.metaqtl. MapCluster 
  
   setMaxClusterNumber(int)   - 
Method in class org.metaqtl.algo. QtlClustAlgorithm 
  
   setMetaQtls(Qtl[])   - 
Method in class org.metaqtl. Chromosome 
  
   setModel(int, int[])   - 
Method in class org.metaqtl. MetaQtlModel 
  
   setMrkThresh(int)   - 
Method in class org.metaqtl. ChromCluster 
  
   setMrkThreshold(int)   - 
Method in class org.metaqtl.algo. MetaMapAlgorithm 
  
   setName(String)   - 
Method in class org.metaqtl.bio.entity.bean. BioEntityBean 
  
   setName(String)   - 
Method in class org.metaqtl.bio.entity.bean. PropertyBean 
  
   setName(String)   - 
Method in class org.metaqtl.bio.entity. BioEntity 
  
   setName(String)   - 
Method in class org.metaqtl.bio.entity. BioEntityCollection 
  
   setName(String)   - 
Method in interface org.metaqtl.bio. IBioEntity 
  
   setParameter(String)   - 
Static method in class org.metaqtl.main. MetaMain 
  
   setParentTerm(IBioOntologyTerm)   - 
Method in class org.metaqtl.bio.entity. OntologyTerm 
 Sets the parent of the term.
   setPedigree(IBioIndividualTree)   - 
Method in class org.metaqtl.bio.entity. Individual 
  
   setPopulation(IBioPopulation)   - 
Method in class org.metaqtl.bio.entity. Individual 
  
   setPopulation(IBioPopulation)   - 
Method in interface org.metaqtl.bio. IBioIndividual 
  
   setPosition(ILGroupPosition)   - 
Method in class org.metaqtl.bio.entity. AlleleContainer 
  
   setPosition(ILGroupPosition)   - 
Method in class org.metaqtl.bio.entity. Locus 
  
   setPosition(double)   - 
Method in class org.metaqtl.bio.entity. Locus 
 Set the position of the locus on the linkage group in
 static mode i.e without uncertainty.
   setPosition(double, double)   - 
Method in class org.metaqtl.bio.entity. Locus 
 Set the position of the locus on the linkage group in
 estimate mode i.e with uncertainty.
   setPosition(ILGroupPosition)   - 
Method in interface org.metaqtl.bio. IBioLocus 
  
   setPosition(double)   - 
Method in interface org.metaqtl.bio. IBioLocus 
  
   setPosition(double, double)   - 
Method in interface org.metaqtl.bio. IBioLocus 
  
   setProba(double[][])   - 
Method in class org.metaqtl. Chromosome 
  
   setProperties(ArrayList)   - 
Method in class org.metaqtl.bio.entity.bean. BioEntityBean 
  
   setProperties(Properties)   - 
Method in class org.metaqtl.bio.entity. BioEntity 
  
   setProperties(Properties)   - 
Method in class org.metaqtl.bio.entity. BioEntityCollection 
  
   setProperties(Properties)   - 
Method in interface org.metaqtl.bio. IBioEntity 
 Sets the properties of the entity.
   setPvalue(double)   - 
Method in class org.metaqtl.algo. QtlProjAlgorithm 
  
   setQtlIdx(int[])   - 
Method in class org.metaqtl. MetaQtlResult 
 Set the indices of the Qtl which are implied in
 this result.
   setRatio(double)   - 
Method in class org.metaqtl.algo. QtlProjAlgorithm 
  
   setRoot(IBioOntologyTerm)   - 
Method in class org.metaqtl.bio.entity. Ontology 
  
   setRoot(OntologyTerm)   - 
Method in class org.metaqtl.bio.entity. Ontology 
  
   setRoot(IBioOntologyTerm)   - 
Method in interface org.metaqtl.bio. IBioOntology 
 Sets the root of the ontology.
   setSD(Double[])   - 
Method in class org.metaqtl. MetaQtlResult 
 Set the standard deviations of the Qtl.
   setSize(int)   - 
Method in class org.metaqtl.bio.entity. Population 
 Set the population size.
   setTerm(String, List)   - 
Method in class org.metaqtl. MetaDico 
  
   setToken(String)   - 
Method in class org.metaqtl.bio.util. TabulatedReader 
  
   setToken(String)   - 
Method in class org.metaqtl.bio.util. TabulatedWriter 
  
   setTraitNames(int, String[])   - 
Method in class org.metaqtl. MetaQtlModel 
  
   setTree(Tree)   - 
Method in class org.metaqtl. MetaQtlResult 
  
   setTree(Tree)   - 
Method in class org.metaqtl. QtlPartition 
  
   setTreeMethod(int)   - 
Method in class org.metaqtl.algo. QtlTreeAlgorithm 
  
   setValue(String)   - 
Method in class org.metaqtl.bio.entity.bean. PropertyBean 
  
   setX(double)   - 
Method in class org.metaqtl.graph. AnchoredElement 
  
   setX(double)   - 
Method in class org.metaqtl.graph. ChromAxe 
  
   setX(Double[])   - 
Method in class org.metaqtl. MetaQtlResult 
 Set the observed positions of the Qtl.
   setX(double)   - 
Method in class org.metaqtl. TreeNode 
  
   setY(double)   - 
Method in class org.metaqtl.graph. AnchoredElement 
  
   setY(double)   - 
Method in class org.metaqtl. TreeNode 
  
   setYMax(double)   - 
Method in class org.metaqtl.graph. ChromAxe 
  
   setYMin(double)   - 
Method in class org.metaqtl.graph. ChromAxe 
  
   SF   - 
Static variable in interface org.metaqtl.bio. IBioCrossTypes 
  
   shareFlanking   - 
Variable in class org.metaqtl. Qtl.QTLProj 
 True if the two maps share a common marker sequence
 around the QTL.
   shortForm()   - 
Method in class org.metaqtl.main. CmdLineParser.Option 
  
   SIGN(double, double)   - 
Static method in class org.metaqtl.numrec. NumericalUtilities 
 Returns abs(a) if b > 0, -abs(a) otherwise.
   SINGLE_COMMON   - 
Static variable in class org.metaqtl.graph. ChromLayer 
  
   SINGLE_COMMON_COLOR   - 
Static variable in class org.metaqtl.graph. MetaGraphPar 
  
   size   - 
Variable in class org.metaqtl.bio.entity. Population 
  
   size()   - 
Method in class org.metaqtl.bio.util. TabulatedReader 
 Returns the number of keys, i.e the number of columns in the file.
   size()   - 
Method in class org.metaqtl.bio.util. TabulatedWriter 
  
   size   - 
Variable in class org.metaqtl. Qtl.QTLCross 
 The number of individuals.
   skeleton   - 
Variable in class org.metaqtl. Chromosome 
 The skeleton status for this member
   sortCluster()   - 
Method in class org.metaqtl. EMResult 
 Sort the components in increasing order.
   sortLociByPosition(IBioLocus[])   - 
Static method in class org.metaqtl.bio.util. BioUtilities 
 Sort tab with loci
   SQR(double)   - 
Static method in class org.metaqtl.numrec. NumericalUtilities 
 Returns a^2
   standardError()   - 
Method in interface org.metaqtl.bio.util. ILGroupPosition 
  
   standardError()   - 
Method in class org.metaqtl.bio.util. LGroupPosition 
  
   STATIC   - 
Static variable in interface org.metaqtl.bio.util. ILGroupPosition 
  
   StaticLocusPosition   - Class in  org.metaqtl.bio.util  Class Description Here   StaticLocusPosition(double)   - 
Constructor for class org.metaqtl.bio.util. StaticLocusPosition 
  
   stats   - 
Variable in class org.metaqtl. Qtl 
 The QTL statistics
   status   - 
Variable in class org.metaqtl.graph. ChromLayer.CMrkTickPath 
  
   status   - 
Variable in class org.metaqtl.graph. ChromLayer.CMrkTickPoint 
  
   stderr   - 
Variable in class org.metaqtl.bio.entity.bean. LocusBean 
  
   stderr   - 
Variable in class org.metaqtl.bio.util. LGroupPosition 
  
   string2color(String)   - 
Static method in class org.metaqtl.graph. MetaGraphPar 
  
   string2font(String)   - 
Static method in class org.metaqtl.graph. MetaGraphPar 
  
   summarize(OutputStream)   - 
Method in class org.metaqtl. ChromCluster.ClusterGraph 
  
   SVDAlgorithm   - Class in  org.metaqtl.algo  This class defines methods to solve usual linear algebra
 problems by using Singular Value Decomposition (SVD) strategy.   SVDAlgorithm()   - 
Constructor for class org.metaqtl.algo. SVDAlgorithm 
  
   SVDBackSub(double[][], double[], double[][], double[], int, int)   - 
Static method in class org.metaqtl.algo. SVDAlgorithm 
 Solves A.X=B for a vector X, where  A  is specified by the
 matrix  U , V  and the vector  w 
 as returned by the SVDecomposition() method.
   SVDecomposition(double[][], double[][], double[], int, int)   - 
Static method in class org.metaqtl.algo. SVDAlgorithm 
 Given a matrix  A , this routine computes its singular
 value decomposition A = U.W.V' .
   SVDMFit(double[][], double[], double[], int, int)   - 
Static method in class org.metaqtl.algo. SVDAlgorithm 
 Given a set of data points y[1..m] with indiviudal weights
 w[1..m], use chi2 minimization to determine the coefficients a[1..n] of the fitting
 function y = X*a.
   swap   - 
Variable in class org.metaqtl. Qtl.QTLProj 
 True if the flanking marker order are inversed between
 the skeleton and the original map.
   synchChromGraphs(ChromGraph[], Set)   - 
Static method in class org.metaqtl.main. MetaMain 
  
   SYNONYMS   - 
Static variable in interface org.metaqtl.bio. IOntologyTermProperties 
 The synonyms property for a term in a ontology.
 
 
    
 T  
 
   TabulatedReader   - Class in  org.metaqtl.bio.util  This class can be used to parse tabulated file in which the first line
 contains the label of the tabulated fields.   TabulatedReader(Reader)   - 
Constructor for class org.metaqtl.bio.util. TabulatedReader 
  
   TabulatedReader(InputStream)   - 
Constructor for class org.metaqtl.bio.util. TabulatedReader 
  
   TabulatedWriter   - Class in  org.metaqtl.bio.util  General comments must be added here.   TabulatedWriter(OutputStream)   - 
Constructor for class org.metaqtl.bio.util. TabulatedWriter 
  
   TabulatedWriter(Writer)   - 
Constructor for class org.metaqtl.bio.util. TabulatedWriter 
  
   TEXT_WIDTH   - 
Static variable in class org.metaqtl.graph. QtlLayer 
 The default width of the Text attribute of the
 Qtl
   tgidx   - 
Variable in class org.metaqtl. MetaQtlData 
 The indices of the QTL in the trait
 groups.
   tgnames   - 
Variable in class org.metaqtl. MetaQtlData 
 The trait group names.
   theta   - 
Variable in class org.metaqtl. MultiFitResult 
 The parameter estimates.
   TICK_CEX_HEIGHT   - 
Static variable in class org.metaqtl.graph. QtlLayer 
 The default cex parameter for the tick height
 of the Qtl
   TICK_CEX_WIDTH   - 
Static variable in class org.metaqtl.graph. QtlLayer 
 The default cex parameter for the tick width
 of the Qtl
   TINY_MAPPING_DISTANCE   - 
Static variable in interface org.metaqtl. IMetaQtlConstants 
 The minimal distance in morgan
   TINY_MAPPING_VARIANCE   - 
Static variable in interface org.metaqtl. IMetaQtlConstants 
 the minimal variance in morgan
   TINY_Z_PROBA   - 
Static variable in class org.metaqtl.algo. EMAlgorithm 
 The minimal value for Z proba in EM-clustering
   to   - 
Variable in class org.metaqtl.graph. QtlUnit 
  
   to   - 
Variable in class org.metaqtl. Qtl.QTLSupport 
 The position from the first marker 
 to the right border of the IC.
   toChromosome(IBioLGroup, IBioCross, int, int)   - 
Static method in class org.metaqtl.adapter. ChromosomeAdapter 
 This method converts a linkage group into a chromosome of type
   Chromosome   with the given cross design
 and the given mapping context.
   toChromosomes(IBioGenome)   - 
Static method in class org.metaqtl.adapter. ChromosomeAdapter 
 This methods converts the given map
 into an array of   Chromosome   which represents
 the chromosomes of the input mpe.
   toEntity(BioEntityBean, IBioEntity)   - 
Static method in class org.metaqtl.bio.entity.adapter. BioEntityAdapter 
  
   toEntity(Object)   - 
Method in class org.metaqtl.bio.entity.adapter. BioEntityCollectionAdapter 
  
   toEntity(Object)   - 
Method in class org.metaqtl.bio.entity.adapter. GeneticMapBeanAdapter 
  
   toEntity(Object)   - 
Method in class org.metaqtl.bio.entity.adapter. LGroupBeanAdapter 
  
   toEntity(Object)   - 
Method in class org.metaqtl.bio.entity.adapter. LocusBeanAdapter 
  
   toEntity(Object)   - 
Method in class org.metaqtl.bio.entity.adapter. OntologyBeanAdapter 
  
   toEntity(Object)   - 
Method in class org.metaqtl.bio.entity.adapter. OntologyTermBeanAdapter 
  
   toEntity(Object)   - 
Method in interface org.metaqtl.bio. IBioAdapter 
 Convert the given bio entity object into
 another compliant object.
   toIBioGenome(Chromosome[], int)   - 
Static method in class org.metaqtl.adapter. ChromosomeAdapter 
 This routine converts an array of chromosomes into a genetic map
 with as many chromosomes as the length of the array.
   toIBioGenome(MetaMap)   - 
Static method in class org.metaqtl.adapter. MetaMapAdapter 
 This methos convert a  MetaMap  object into
 an object which is consistent with the  IBioGenome 
 interface.
   toQTL(IBioLocus)   - 
Static method in class org.metaqtl.adapter. QtlAdapter 
 This method converts an object which class
 implements the  IBioLocus  interface
 into a   Qtl  .
   toString()   - 
Method in class org.metaqtl.bio.entity. LGroup 
  
   toString()   - 
Method in class org.metaqtl. Chromosome 
  
   toString()   - 
Method in class org.metaqtl. MultiFitResult 
  
   totd   - 
Variable in class org.metaqtl. Chromosome 
 The total distance of the chromosome.
   trait   - 
Variable in class org.metaqtl. MetaQtlResult 
 The name of the trait.
   trait   - 
Variable in class org.metaqtl. Qtl 
 The QTL trait data.
   traitNames   - 
Variable in class org.metaqtl. MetaQtlModel 
 The trait names per chromosome.
   transformY(double)   - 
Method in class org.metaqtl.graph. ChromAxe 
 Do a affine transformation of the given
 position  d  which is assumed
 to be in [0,dmax] in order to have its
 representation in the chromosome axe.
   tree   - 
Variable in class org.metaqtl. MetaQtlResult 
 The qtl tree
   tree   - 
Variable in class org.metaqtl. QtlPartition 
 The Qtl tree.
   Tree   - Class in  org.metaqtl  This class defines a tree.   Tree()   - 
Constructor for class org.metaqtl. Tree 
  
   Tree(TreeNode)   - 
Constructor for class org.metaqtl. Tree 
  
   tree_method   - 
Variable in class org.metaqtl.algo. QtlTreeAlgorithm 
 This global variable defines the method used by the aglomerative
 hierarchical clustering.
   TREE_NODE_WIDTH   - 
Static variable in class org.metaqtl.graph. MetaGraphPar 
  
   TreeFactory   - Class in  org.metaqtl.factory  A factory to deal with distance trees.   TreeFactory()   - 
Constructor for class org.metaqtl.factory. TreeFactory 
  
   TreeNode   - Class in  org.metaqtl  This class represents a node in a binary Tree obtained
 when applying usual hirarchical clustering methods.   TreeNode(int)   - 
Constructor for class org.metaqtl. TreeNode 
 Creates a new instance of  TreeNode 
 with the given indice  idx .
   TreeNode(int, TreeNode[])   - 
Constructor for class org.metaqtl. TreeNode 
 Creates a new instance of   TreeNode  with the
 given indice  idx  and the children of the node.
   TreeNode()   - 
Constructor for class org.metaqtl. TreeNode 
 Creates a new instance of  TreeNode  with
 default attribute values.
   type   - 
Variable in class org.metaqtl.bio.entity.bean. LocusBean 
  
   type   - 
Static variable in class org.metaqtl.bio.util. BioCrossTypes 
  
   type   - 
Variable in class org.metaqtl. Qtl.QTLCross 
 The type of the cross.
   TypesString   - 
Static variable in interface org.metaqtl.bio. IBioLocus 
  
   typeToString(int)   - 
Static method in class org.metaqtl.bio.util. BioLocusTypes 
  
 
 
    
 U  
 
   unit   - 
Variable in class org.metaqtl. Qtl.QTLTrait 
 The unit of the trait.
   UNKNOWN   - 
Static variable in interface org.metaqtl.bio. IBioCrossTypes 
  
   unload(IBioEntity, Writer)   - 
Method in class org.metaqtl.bio.entity.factory. BioEntityFactory 
  
   unload(IBioEntity, OutputStream)   - 
Method in class org.metaqtl.bio.entity.factory. BioEntityFactory 
  
   unload(IBioEntity, Writer)   - 
Method in class org.metaqtl.bio.entity.factory. MapChartFactory 
 TODO (non-Javadoc)
   unload(IBioEntity, OutputStream)   - 
Method in class org.metaqtl.bio.entity.factory. MapChartFactory 
 TODO (non-Javadoc)
   unload(IBioEntity, Writer)   - 
Method in class org.metaqtl.bio.entity.factory. MapMakerFactory 
  
   unload(IBioEntity, OutputStream)   - 
Method in class org.metaqtl.bio.entity.factory. MapMakerFactory 
  
   unload(IBioEntity, Writer)   - 
Method in class org.metaqtl.bio.entity.factory. MapRawFactory 
  
   unload(IBioEntity, OutputStream)   - 
Method in class org.metaqtl.bio.entity.factory. MapRawFactory 
  
   unload(IBioEntity, Writer)   - 
Method in class org.metaqtl.bio.entity.factory. MapTabFactory 
  
   unload(IBioEntity, OutputStream)   - 
Method in class org.metaqtl.bio.entity.factory. MapTabFactory 
  
   unload(IBioEntity, Writer)   - 
Method in class org.metaqtl.bio.entity.factory. OntologyTabFactory 
  
   unload(IBioEntity, OutputStream)   - 
Method in class org.metaqtl.bio.entity.factory. OntologyTabFactory 
  
   unload(IBioEntity, OutputStream)   - 
Method in class org.metaqtl.bio.entity.factory. XmlBioEntityFactory 
  
   unload(IBioEntity, Writer)   - 
Method in class org.metaqtl.bio.entity.factory. XmlBioEntityFactory 
  
   update(Reader)   - 
Static method in class org.metaqtl.factory. MetaGraphParFactory 
  
   updateLeafIdx(int[])   - 
Method in class org.metaqtl. Tree 
  
   updateLoglikelihood(double[], double[], EMResult)   - 
Static method in class org.metaqtl.algo. EMAlgorithm 
 Updates the loglikelihood value.
   updateMappingContext(int, int)   - 
Method in class org.metaqtl. Chromosome 
 Updates the mapping context of the chromosome.
   updateMuVector(double[], double[], EMResult)   - 
Static method in class org.metaqtl.algo. EMAlgorithm 
 Updates the vector of mixture components and returns the new
 values.
   updatePiVector(EMResult)   - 
Static method in class org.metaqtl.algo. EMAlgorithm 
 Updates the vector of mixture mixings and returns the new
 values.
   updateZMatrix(double[], double[], EMResult)   - 
Static method in class org.metaqtl.algo. EMAlgorithm 
 Updates the Z matrix.
 
 
    
 V  
 
   value   - 
Variable in class org.metaqtl.bio.entity.bean. PropertyBean 
  
   varianceDistance(double, double, int, int)   - 
Static method in class org.metaqtl.util. MappingFunction 
 Returns the variance of the distance for a given recombination
 rate r, its variance and the mapping function.
   verbose   - 
Variable in class org.metaqtl.main. MetaMain 
  
   VERSION   - 
Static variable in interface org.metaqtl. IMetaQtlConstants 
 The version of the package.
   VERTICAL_MODE   - 
Static variable in class org.metaqtl. QtlPartition 
  
   virtual   - 
Variable in class org.metaqtl.bio.entity.bean. OntologyTermBean 
 The virual status of the term.
 
 
    
 W  
 
   wantsValue()   - 
Method in class org.metaqtl.main. CmdLineParser.Option 
 Tells whether or not this option wants a value
   WARD_METHOD   - 
Static variable in class org.metaqtl.algo. HClustAlgorithm 
 Ward method
   WardMethod(double[], double[])   - 
Static method in class org.metaqtl.algo. HClustAlgorithm 
 This method performs a hierarchical clustering
 of the given data points using the Ward's method.
   WIDTH   - 
Static variable in class org.metaqtl.graph. GraphFactory 
  
   width   - 
Variable in class org.metaqtl.graph. GraphShape 
  
   width   - 
Variable in class org.metaqtl.graph. Layer 
 The width of the layer.
   width   - 
Variable in class org.metaqtl.graph. QtlUnit 
  
   WITH_CHROM_NAME   - 
Static variable in class org.metaqtl.graph. MetaGraphPar 
  
   WITH_COMMON_MARKER   - 
Static variable in class org.metaqtl.graph. MetaGraphPar 
  
   WITH_LEGEND   - 
Static variable in class org.metaqtl.graph. MetaGraphPar 
  
   WITH_MAP_NAME   - 
Static variable in class org.metaqtl.graph. MetaGraphPar 
  
   WITH_MARKER_NAME   - 
Static variable in class org.metaqtl.graph. MetaGraphPar 
  
   WITH_MARKER_POSITION   - 
Static variable in class org.metaqtl.graph. MetaGraphPar 
  
   WITH_QTL_NAME   - 
Static variable in class org.metaqtl.graph. MetaGraphPar 
 Add the Qtl name.
   workAmount   - 
Variable in class org.metaqtl.algo. MetaAlgorithm 
 The amount of work to do.
   workProgress   - 
Variable in class org.metaqtl.algo. MetaAlgorithm 
 The amount of work done.
   write(EMResult, OutputStream)   - 
Static method in class org.metaqtl.factory. EMResultFactory 
  
   write(EMResult, PrintWriter)   - 
Static method in class org.metaqtl.factory. EMResultFactory 
  
   write(OutputStream)   - 
Static method in class org.metaqtl.factory. MetaGraphParFactory 
  
   write(Writer)   - 
Static method in class org.metaqtl.factory. MetaGraphParFactory 
  
   write(MetaMap, OutputStream)   - 
Static method in class org.metaqtl.factory. MetaMapSummaryFactory 
 This methods writes out the summary for a consensus genetic map into
 a given output stream  stream .
   write(MetaQtlAnalysis, OutputStream)   - 
Static method in class org.metaqtl.factory. MetaQtlAnalysisFactory 
 This methods writes out in the given  stream  the results
 of a QTL meta-analysis.
   write(MetaQtlAnalysis, OutputStream)   - 
Static method in class org.metaqtl.factory. MetaQtlAnalysisModelFactory 
  
   write(MetaQtlAnalysis, Writer)   - 
Static method in class org.metaqtl.factory. MetaQtlAnalysisModelFactory 
  
   write(MetaQtlAnalysis, OutputStream)   - 
Static method in class org.metaqtl.factory. MetaQtlAnalysisSummaryFactory 
 This method writes out the summary for a givene QTL meta-analysis result
  analysis  on the given output stream  stream .
   write(MetaQtlModel, OutputStream)   - 
Static method in class org.metaqtl.factory. MetaQtlModelFactory 
  
   write(MetaQtlModel, Writer)   - 
Static method in class org.metaqtl.factory. MetaQtlModelFactory 
  
   write(Tree, OutputStream)   - 
Static method in class org.metaqtl.factory. TreeFactory 
 This method writes out the given tree  nodes  as an array of 
   TreeNode   on the given output stream  stream .
   write(Tree, PrintWriter)   - 
Static method in class org.metaqtl.factory. TreeFactory 
 This method writes out the given tree  nodes  as an array of 
   TreeNode   using the given writer  writer .
   writeImg(ChromGraph[], File, String)   - 
Static method in class org.metaqtl.main. MetaMain 
  
   writeMap(IBioGenome, String, String)   - 
Static method in class org.metaqtl.main. MetaMain 
  
   writeMap(IBioGenome, String)   - 
Static method in class org.metaqtl.main. MetaMain 
  
   writePar(File)   - 
Static method in class org.metaqtl.main. MetaMain 
  
 
 
    
 X  
 
   x   - 
Variable in class org.metaqtl.graph. AnchoredElement 
  
   x   - 
Variable in class org.metaqtl.graph. GraphShape 
  
   x   - 
Variable in class org.metaqtl.graph. QtlUnit 
  
   x   - 
Variable in class org.metaqtl. MetaQtlResult 
 The QTL positions.
   x   - 
Variable in class org.metaqtl. TreeNode 
  
   xc   - 
Variable in class org.metaqtl. TreeNode 
  
   Xml2A   - Class in  org.metaqtl.main      Xml2A()   - 
Constructor for class org.metaqtl.main. Xml2A 
  
   XmlBioEntitiesFactory   - Class in  org.metaqtl.bio.entity.factory  Class Description Here   XmlBioEntitiesFactory()   - 
Constructor for class org.metaqtl.bio.entity.factory. XmlBioEntitiesFactory 
  
   XmlBioEntityFactory   - Class in  org.metaqtl.bio.entity.factory  Class Description Here   XmlBioEntityFactory()   - 
Constructor for class org.metaqtl.bio.entity.factory. XmlBioEntityFactory 
  
   XmlGeneticMapFactory   - Class in  org.metaqtl.bio.entity.factory  Class Description Here   XmlGeneticMapFactory()   - 
Constructor for class org.metaqtl.bio.entity.factory. XmlGeneticMapFactory 
  
   XmlOntologyFactory   - Class in  org.metaqtl.bio.entity.factory      XmlOntologyFactory()   - 
Constructor for class org.metaqtl.bio.entity.factory. XmlOntologyFactory 
  
 
 
    
 Y  
 
   y   - 
Variable in class org.metaqtl.graph. AnchoredElement 
  
   y   - 
Variable in class org.metaqtl.graph. GraphShape 
  
   y   - 
Variable in class org.metaqtl. TreeNode 
  
   y_from   - 
Variable in class org.metaqtl.graph. QtlShape 
  
   y_pos   - 
Variable in class org.metaqtl.graph. QtlShape 
  
 
 
    
 Z  
 
   z   - 
Variable in class org.metaqtl. EMResult 
 The cluster membership probabilties.
 
 
 A   B   C   D   E   F   G   H   I   K   L   M   N   O   P   Q   R   S   T   U   V   W   X   Y   Z  

 
   
  
 
 
 
   
 
   
          Overview     
        Package   
        Class   
        Use   
          Tree     
          Deprecated     
       Index    
          Help     
   
 
 
  
 
 
 

 
  
 PREV 
 NEXT  
  
    FRAMES     
   NO FRAMES     
  
    All Classes  ');
  }
  //-->
 
 
    All Classes
